# Complex associations between cancer progression and immune gene expression reveals early influence of transmissible cancer on Tasmanian devils

**DOI:** 10.3389/fimmu.2024.1286352

**Published:** 2024-03-07

**Authors:** Nynke Raven, Marcel Klaassen, Thomas Madsen, Menna Jones, David G. Hamilton, Manuel Ruiz-Aravena, Frederic Thomas, Rodrigo K. Hamede, Beata Ujvari

**Affiliations:** ^1^ Deakin University, School of Life and Environmental Sciences, Centre for Integrative Ecology, Geelong, VIC, Australia; ^2^ School of Natural Sciences, University of Tasmania, Hobart, TAS, Australia; ^3^ Mississippi State University, Forest & Wildlife Research Center (FWRC)-Wildlife, Fisheries & Aquaculture, Starkville, MS, United States; ^4^ CREEC/CANECEV, CREES-MIVEGEC, Univ. Montpellier, CNRS, IRD, Montpellier, France

**Keywords:** tumor progression, immune system, marsupial, infectious disease, life-history traits, regression, resistance, tolerance

## Abstract

The world’s largest extant carnivorous marsupial, the Tasmanian devil, is challenged by Devil Facial Tumor Disease (DFTD), a fatal, clonally transmitted cancer. In two decades, DFTD has spread across 95% of the species distributional range. A previous study has shown that factors such as season, geographic location, and infection with DFTD can impact the expression of immune genes in Tasmanian devils. To date, no study has investigated within-individual immune gene expression changes prior to and throughout the course of DFTD infection. To explore possible changes in immune response, we investigated four locations across Tasmania that differed in DFTD exposure history, ranging between 2 and >30 years. Our study demonstrated considerable complexity in the immune responses to DFTD. The same factors (sex, age, season, location and DFTD infection) affected immune gene expression both across and within devils, although seasonal and location specific variations were diminished in DFTD affected devils. We also found that expression of both adaptive and innate immune genes starts to alter early in DFTD infection and continues to change as DFTD progresses. A novel finding was that the lower expression of immune genes MHC-II, NKG2D and CD8 may predict susceptibility to earlier DFTD infection. A case study of a single devil with regressed tumor showed opposite/contrasting immune gene expression patterns compared to the general trends observed across devils with DFTD infection. Our study highlights the complexity of DFTD’s interactions with the host immune system and the need for long-term studies to fully understand how DFTD alters the evolutionary trajectory of devil immunity.

## 1 Introduction

Transmissible cancers are a rare type of pathogen, where the cancer cells themselves are contagious. So far, eleven transmissible cancers have been identified, in bivalves, dogs and Tasmanian devils (1). The Tasmanian devil is the largest marsupial carnivore endemic to the island state of Tasmania. Over the past 30 years, their total population has decreased by 68% and locally by 82% due to a transmissible cancer, Devil Facial Tumor Disease (DFTD) (2). The cancer was first documented in 1996, in the northeast of the state, but most likely originated ~10 years earlier (3). This disease predominantly spreads through devils biting each other, e.g. during mating and feeding interactions (4) and has since spread through Tasmania (2). While DFTD is still fatal in most cases, devils are showing signs of adaptation to the disease. Some studies detected changes in allele frequency in cancer and immune system associated genes within 4-6 generations after disease emergence (5, 6), while others (7) detected no significant difference in genome-wide SNPs across the devil’s range. Moreover, reduction in reproductive age (8), changes in how long animals survive with DFTD (9) and even complete tumor regressions have been documented (10–12).

Increasing incidences of tumor regressions (Hamede, pers comm) indicate that the devils are able to mount immune and tumor suppressor responses to DFTD. Indeed, some devils with complete regressed tumors had detectable serum antibodies, while in one animal tumor infiltrating T lymphocytes were observed (10). Differences in genomic regions between individuals where tumors regressed and did not regress have been observed (12). In cases where tumors regressed, allele frequency changes were detected in genes possibly associated with angiogenesis in the tumor microenvironment [PAX3 and TLL1 (11)]. Closer examination of the tumors showed stimulation of the RAS pathway in regressed tumors, whereas the RASL11A gene was silenced in non-regressed tumors (13). Despite the positive outcomes observed in some devils with regressed tumors, disease outcomes are more complex as some regressed animals became reinfected after suppressing and eliminating the initial tumors (10). The presence of tumor regressions in populations suggests that some devils are able to mount immune responses to DFTD, indicating the existence of a resistance phenotype. However, there is also evidence that devils are tolerating, rather than actively resisting DFTD (9, 14). When tolerating a pathogen, immunopathology is downregulated and there is no direct immune response, with the negative impact of the infection instead being buffered and controlled (15, 16). Resistance to a pathogen as a defense mechanism involves immunopathology, which can include activating adaptive immune genes like T and B cells (17), which the host uses to directly attack the pathogen (18). It is possible that both resistance and tolerance are involved in the Tasmanian devils’ response to DFTD (19).

Tasmanian devil responses to DFTD infection are complex, with no differences between the transcriptomes of healthy and DFTD infected animals detected in lip tissue (20). However, more targeted analysis from blood has shown shifts in immune gene expression with DFTD infection (21). Innate immune gene expression (particularly CD16) increased, and some genes associated with adaptive immune function were downregulated with DFTD infection (21). Targeted sequencing also showed a decline in T-cell receptor diversity and restricted T-cell clonal expansion (22). Blood cell counts identified an increase in white blood cell numbers, neutrophils, and platelets, and concentration of fibrinogen (blood protein assisting with clotting) with DFTD infection, as well as decreases in lymphocytes erythrocyte, and hemoglobin concentrations (23). Shorter telomere length (24, 25) and lower IgM : IgG antibody ratio (26) have been associated with DFTD susceptibility. DFTD infection can also alter gene expression within populations, as the DFTD arrival to individual populations was associated with a decrease of single nucleotide polymorphism (SNPs) in genomic regions connected with environment and abiotic factors (27). These studies show the complexity of immune responses to DFTD and demonstrate the need for a variety of approaches to understand the impact of DFTD infection on Tasmanian devil immune function.

Much of the previous work has been based on cross-sectional data (sampling across individuals), and not necessarily reflects how expression of immune genes changes over time within individuals (28). For example, (14) used repeat measures and identified that the cost of DFTD infection on body condition of individual devils was dependent on both the sex of the devil and the relative size of the tumor. Here, we use a combination of cross-sectional and longitudinal data, including samples from devils both before and after evidence of DFTD infection. We targeted ten previously published immune genes, which are expressed only in specific immune cell types and that increases or decreases in expression likely reflect shifts in the frequency of those specific immune cells as well as key functions of the cell types/genes measured (see detailed description in (21) and **Supplementary Table S1** in the **Supplementary Material**). With targeting these genes we aimed to understand how host immune gene expression changes with DFTD progression. First, we predicted sex, age, season, location and DFTD infection would have a similar impact on gene expression both within and between individuals. Second, we investigated how gene expression changes with DFTD progression. Thirdly, we predicted that animals with lower expression of immune genes as juveniles may have a predisposition to DFTD. Finally, as we had managed to recapture a female devil multiple times, and to follow her through tumor progression from tumor emergence to complete regression, we used this animal as a case study, to determine if there is any immune gene expression difference in the regressed animal, compared to the general population of Tasmanian devils.

## 2 Methods

**Figure 1:**
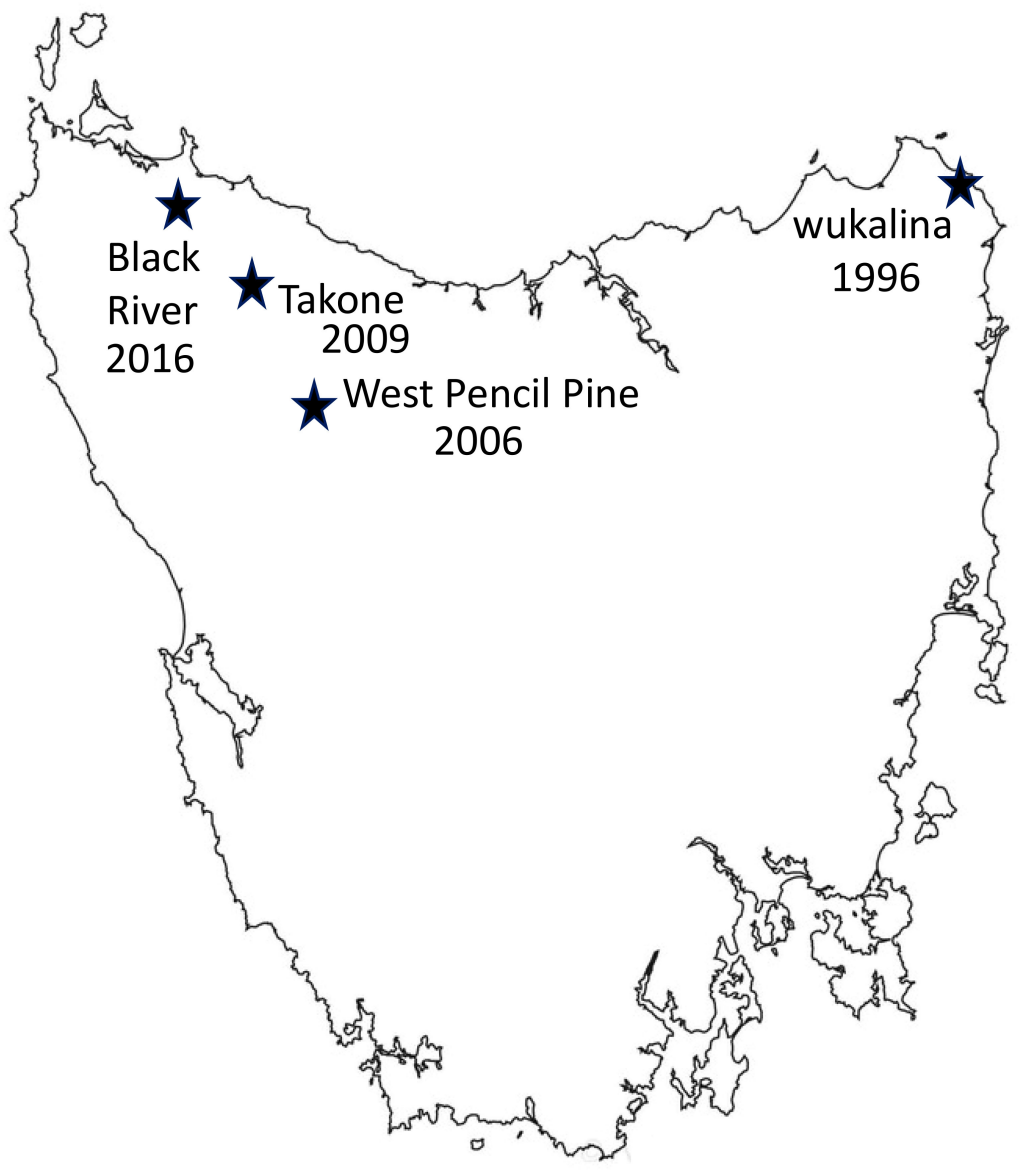
Map showing four sampling locations in Tasmania, along with the year when Devil Facial Tumor Disease (DFTD) was first detected in each location. Number of years DFTD has been present in each location: wukalina 27 years, West Pencil Pine 17 years, Takone 14 years, Black river 7 years.

Fieldwork was undertaken at four sites in Tasmania, wukalina, West Pencil Pine (WPP), Takone and Black River (BRI) (**Figure 1**), between November 2016 and May 2022. Blood samples used in this study were collected up until February 2020, and follow-up metadata about disease outcomes was available until May 2022. Sampling occurred every three months to coincide with the life history stages of the Tasmanian devils: including just prior to the breeding season (February), pregnancy or small pouch young (May), large young either in the pouch or dens (August) and young devils becoming independent (November). Devils were microchipped during first capture and therefore could be identified at future recaptures. The devils are aged based on their dental wear as described in (4).

Across the four locations a total of 605 samples were collected from 375 individuals. 140 individuals were captured more than once, making up 370 of the total samples, while the remaining 235 individuals were only captured a single time. Of these recaptures, 62 individuals had samples taken both when they appeared healthy and when they had visible DFTD tumors (**Table 1**). A different subset of the data was used for each analysis, which are described with the relevant analysis method below.

**Table 1:** Number of individual devils recaptured by location.

	Number of individuals captured:			No. of pre and post DFTD infection
	Twice	Three times	>Three times	
Black River	5	11	3	7
West Pencil Pine	21	14	4	18
Takone	21	12	5	19
wukalina	26	14	4	18

Blood samples were collected via peripheral ear vein puncture, with ~0.5mL of blood collected into RNA protect blood tubes (Qiagen, Germantown, MD, USA). Samples were stored immediately in liquid nitrogen until they could be moved to a -80°C freezer.

Sample collection was carried out according to guidelines and regulations approved by University of Tasmania’s Animal Ethics Committee (approval permit A0013326; A0016789) and Deakin University’s Animal Ethics Committee (approval number AEX03-2017). Sampling permit was issued by the Department of Primary Industries, Parks Water and the Environment.

The RNeasy Protect Animal Blood Kit (Qiagen, Germantown, USA) was used for RNA extraction, following manufacturer’s protocol with a slight change in step 17, which was repeated twice. The eluate was directly pipetted back onto the spin column in the repeated step to increase final RNA concentration. The quality and concentration of RNA was determined with the Agilent Bioanalyzer 2100 (Agilent Technologies, Santa Clara, CA) following manufacturer’s protocols and only samples with a RIN of ≥7 were included in the downstream analyses. RNA reverse transcription to cDNA was completed with the Bio-Rad iScript™ Advanced cDNA Synthesis Kit for RT-qPCR (BioRad, California, USA) at 42°C for 30 minutes, and the reaction was inactivated at 85°C for 5 minutes. Previously published primers were used from Raven et al. (21). RT-qPCR reactions were conducted in a BioRad CFX Connect Real-Time System (BioRad, California, USA) machine. Changes in mRNA levels between samples and across plates were quantitated with standard curves, that were generated using 1:5 dilution series. No individual sample Cq values fell outside the standard curves. All standard curves had an R^2^ > 0.99 and RT-qPCR efficiencies ranged between 92.4% and 108%, as per standard procedure (29). RT-qPCR reactions contained 7.5µl of BioRad SsoAdvanced™ Universal SYBR^®^ Green Super Mix (BioRad, California, USA), 0.5µM forward and reverse primers and 1µL of cDNA. RT-qPCR reactions were run at 95°C for 30sec denaturing and 45 cycles of 95°C for 10 sec and 60°C for 30sec (annealing temperature). Fluorescence signal was acquired at the annealing temperature and a final melting curve analysis (from 65°C up to 99°C) was added under continuous fluorescence measurements.

### 2.1 Statistical analysis

All statistical analyses were conducted in R v3.6.1 (30).

#### 2.1.1 Comparing cross-sectional and longitudinal immune gene expression

A previous cross-sectional study on a different dataset, limited to WPP and with each individual measured only once, showed that immune gene expression in Tasmanian devils is influenced by multiple factors with some patterns being general across all devils, whilst others are season specific (21). To determine if the same factors emerging from the cross-sectional dataset also influence immune gene expression within individual devils in the current longitudinal dataset, we used the explanatory variables in the ‘final gene models’ run using the method from Raven et al. (21) with location as an additional explanatory variable to predict gene expressions for our current dataset using the ‘predict’ function in the Car package v3.1-0 (31). Next, for each gene in the longitudinal dataset we compared the predicted gene expressions with the actual gene expression values using four different models. An ordinary linear model with no random effects (0) and three different models including random effects using linear mixed models in the lme4 package 1.1-26 (32). One of these was with a random intercept (1), the other with a random slope (2) and finally one with both a random intercept and a random slope (3). Of these four, the model with the lowest Akaike Information criterion (AICc) was retained and the slope of the resulting relationship was tested against 0 (no relationship) and 1 (identical outcomes of both models). If the cross-sectional models adequately predict gene expression in the longitudinal dataset, the slope should differ from 0 and not significantly differ from 1.

#### 2.1.2 Factors affecting immune gene expression in DFTD affected devils

To determine the factors influencing immune gene expression when animals are infected with DFTD, a subset of the data including only animals with confirmed DFTD tumors was used. Only a single capture from each animal was included. If animals had more than one DFTD infected capture, a single sample was chosen from these animals. When selecting the single sample, we aimed to optimize the even distribution of tumor volume data, resulting in a total of 115 samples from a total of 139 samples. For each gene, we investigated variation in gene expression using a linear model with season, sex, location, age and tumor volume as explanatory variables. There are no techniques to detect latency or metastases, so tumor volume was used as a proxy for disease progression, consistent with previous devil literature (9, 33–36). Tumor volume was calculated using an ellipsoid volume formula (as in 14) from the length, width, and depth measurements of each tumor in mm, summed in the case of multiple tumors. The tumor volume measurements were normalized using the natural log. Since the expression of immune genes can be correlated, the analysis was run individually on each gene. To determine which variables were important for each gene in DFTD-affected animals, all explanatory variables of interest (listed previously) and logical interactions (season x age and tumor volume x age excluded) were included in the full model, to be analyzed using model selection. The ‘dredge’ function within the MuMIn 1.46.0 package (37) was used to evaluate all possible models, respecting marginality constraints, each model was ranked and weighted according to AICc adjusted for sample size. Models within 2 AICc units were retained for further analyses and the model weights adjusted accordingly. An ‘importance’ value was calculated for each explanatory value in each gene model by summing the adjusted model weights. ‘Importance’ values range between 0 (not in any models) and 1 (in every model). A final average model for each gene was created including all explanatory variables with ‘importance’ values of >0.3.

To determine similarities in the relationships with explanatory variables across genes, the importance values were clustered and visualized in a heatmap created using the gplots v 3.1.3 (38) and plotrix v 3.8-2 (39) packages. Similar heatmaps were used to visualize the slopes of each final gene model, which shows the effects of each variable on gene expression (output for final gene models in **Supplementary Table S2**). Marginal-effects means were calculated to display the effect of a specific explanatory variable, accounting for all other variables in the model, using the ggeffects v 1.1.2 package (40). To test for significant differences between the categories, *post-hoc* tests with Sidak adjusted p-values were calculated using emmeans v 1.7.5 (41) and multcomp 1.4-19 (42) packages. Marginal means data with *post-hoc* test outputs in the form of letters were plotted using the ggplots2 v 3.3.6 (43) and patchwork v 1.1.1 packages (44).

#### 2.1.3 Immune gene expression patterns prior to and after DFTD infection

To investigate how immune gene expression changes within an individual devil when they first acquire DFTD, samples from devils captured both when healthy and then recaptured with DFTD were used (62 in total, 33 females and 28 males). To minimize other influences on immune gene expression, only two samples per animal were used: one from the last capture when the devil was healthy and one from the first recapture with detectable DFTD. The difference in the expression of specific genes between the two measurements was determined using a paired T-test, using the rstatix v 0.7.0 (45) and ggpubr v 0.4.0 (46) packages.

#### 2.1.4 Can immune gene expression patterns in juvenile devils predict DFTD susceptibility?

To investigate whether animals that develop DFTD at an early age have a different immune gene expression profile compared with those who develop DFTD later or not at all during their life, the immune gene expression pattern of juvenile devils (11-20 months) with no signs of DFTD were investigated. Animals in this age bracket were categorized based on whether they were DFTD positive (Pos26) or negative (Neg26) by 26 months of age. The Pos26 category contained 23 females and 22 males, while the Neg26 category contained 21 females and 17 males. Animals that were never captured as healthy juvenile and could not be classified into either category were excluded from the analysis. The cut off age was chosen as by this point devils have gone through at least one mating season and should have been exposed to DFTD (47, 48). Devils from each location were included in the analysis; wukalina n=21, BRI n=12, WPP n=22 and Takone n=28. Linear models were created for each gene used to understand the relationship between immune gene expression and DFTD susceptibility. As both location and season have been shown to be important in the expression of the studied genes, these were accounted for in the model (based off analysis number 2). The marginal means for significant results for the variable of interest were plotted.

#### 2.1.5 Immune gene expression variation throughout tumor growth and regression in a single devil

Finally, we had access to a unique dataset of a female devil recaptured seven times with samples from before DFTD infection, during DFTD infection and regression and post DFTD infection. Therefore, we investigated how immune gene expressions change in this female devil over the course of DFTD infection and tumor regression. Immune gene expression patterns from the seven captures were graphed and the trends observed in this single animal were compared to those obtained in the previous analyses (analyses 1-4). Packages used for graphing; ggplots2 v 3.3.6 (43), Tidyverse v 1.3.1 (49), lubridate v 1.8.0 (50). No statistical analysis was used as we only had samples from multiple recaptures available for this single animal.

## 3 Results

### 3.1 Comparing cross-sectional and longitudinal immune gene expression

When cross-sectional models yield similar predictions to the longitudinal models based on the dataset used here, we would predict a slope of 1 between the predicted values of both models. Indeed, for most immune genes, the relationship between the predicted variables obtained from the cross-sectional models and the longitudinal models were not significantly different (*i.e.* the slope between both was not significantly different from 1 while it was significantly different from 0; **Table 2**). The lack of significant difference between the cross-sectional and longitudinal models indicates that the same factors influence most immune gene expressions in a similar fashion within individual devils as between animals. The two exceptions that did not confirm to this general trend were MHC-II and the IgM : IgG ratio. For both MHC-II and the IgM : IgG ratio the slopes were still positive but not significantly different from 0. Moreover, the slope for the IgM : IgG ratio was also significantly different from 1.

**Table 2:** Predicted vs actual gene expression*.

Gene	AIC: no random effects	AIC: random effects	Top random effect model	Models in 2 AIC	Intercept	SE	p-value	Slope	SE	p-value (0)	p-value (1)	R2 cond.	R2 marg.
**IgG**	193.66	195.66	1	0,1	0.09	0.12	0.48	0.83	0.21	**0.00**	0.65	0.20	0.19
**IgM**	214.59	213.77	2	2,3,0	-0.28	0.14	0.05	0.88	0.24	**0.00**	0.64	0.55	0.29
**IgA**	187.50	187.15	1	0,1	-0.09	0.14	0.53	0.67	0.19	**0.00**	0.09	0.32	0.18
**IgE**	227.66	228.45	1	0,1	-0.14	0.20	0.47	0.68	0.19	**0.00**	0.10	0.28	0.16
**CD4**	163.54	165.54	1	0,1	-0.08	0.13	0.56	0.80	0.17	**0.00**	0.23	NA	0.26
**CD8**	183.19	185.12	1	0,1	0.04	0.14	0.77	0.67	0.29	**0.02**	0.26	0.11	0.07
**CD11**	156.39	158.39	1	0,1	-0.07	0.09	0.40	0.63	0.19	**0.00**	**0.05**	NA	0.14
**CD16**	151.61	153.17	1	0,1	0.03	0.10	0.77	0.77	0.15	**0.00**	0.13	0.35	0.29
**MHC-II**	223.48	225.48	1	0,1	-0.31	0.24	0.20	0.40	0.34	0.25	0.08	NA	0.02
**NKG2D**	209.60	210.51	1	0,1	-0.24	0.18	0.18	0.65	0.25	**0.01**	0.17	0.20	0.09
**IgM : IgG**	163.89	165.90	1	0,1	-0.42	0.15	0.01	0.29	0.25	0.24	**0.01**	NA	0.02
**CD4:CD8**	136.43	137.10	1	0,1	-0.03	0.11	0.79	1.12	0.22	**0.00**	0.60	0.43	0.32

*Table shows results for each gene model. Each model was run using four different model types: 0, no random effects, 1, random intercept, 2, random slope, 3, both random intercept and random slope. Akaike Information Criterion (AIC) was reported for model 0, as well as the best fitting random effect model. All models within the top 2 AIC are reported. For the best fitting random effect model, the intercept, slope, standard errors (SE), p-value and R^2^ values are reported. Each top model was rerun to test if the relationship between the predicted and actual gene expression was significantly different from 1 and 0, with p-values for both analyses reported. Bold indicates significant values.

### 3.2 Factors affecting immune gene expression in DFTD positive devils

**Figure 2:**
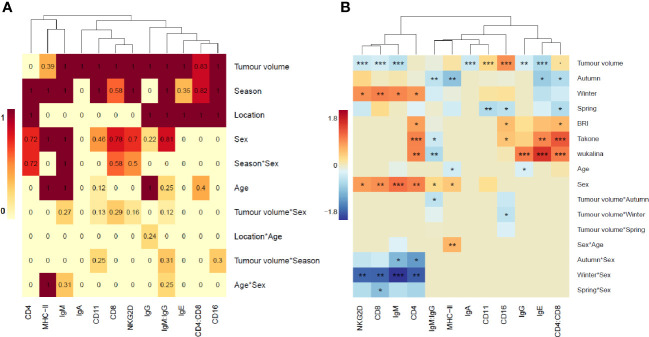
Heatmaps of importance values and model slopes. **(A)** Heatmap as a visual representation of the importance values, calculated from model selection analysis, for each explanatory variable in each gene model. Importance values are the sum of model weights containing each explanatory variable in the top 2 Akaike Information Criterion (AICc) of model selection analysis. Intensity of color increases with increasing importance value. **(B)** Heatmap of the model estimate (slope) for each explanatory variable in each final gene expression model. Orange=positive, blue=negative, intensity of color represents strength of the effect. P-values: ‘***’ <0.001, ‘**’ <0.01, ‘*’<0.05, ‘.’<0.1.

To select variables for the individual gene models, we first investigated the importance of each explanatory variable (season, location, sex, age, and tumor volume and logical interactions) in influencing the expression level of a given gene (**Figure 2A**). Tumor volume was important in 11/12 models, season in 10/12 and location in half of the models. Sex and age were important to some degree in 8/12 and 6/12 models respectively. Overall, the interactions had lower importance in the gene models compared to main effects. All final model outputs are presented in **Supplementary Table S2**.

When running the final linear models on each gene (**Figure 2B**) we found that tumor volume was significant in 9/12 models (p-value >0.001 in 7/12 models). Season as a main effect or as an interaction was significant in 10/12 models, location was significant in all 6 models where it was included. All the genes expressed on T-cells (CD4, CD8 and NKG2D) formed a cluster with IgM, showing similar influences on gene expression and these were the only genes to show a significant interaction with season and sex. Two other immunoglobulins grouped with the CD4:CD8 ratio, showing similar influences of gene expression, and the remaining immunoglobulin grouped with the innate immune genes, MHC-II and the IgM : IgG ratio.

**Figure 3:**
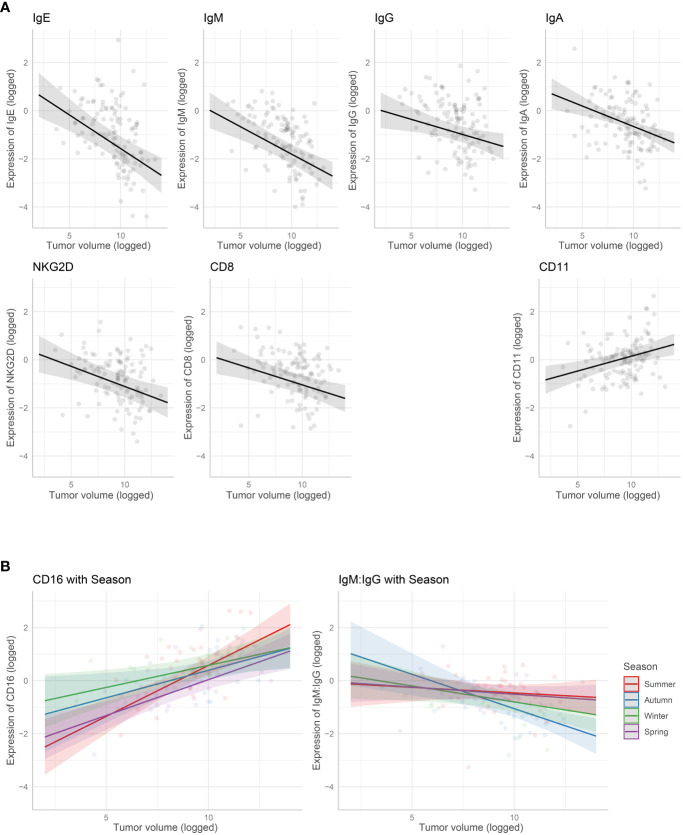
Marginal means plots showing the effect of gene expression with tumor volume. **(A)** Tumour volume as a main effect. **(B)** Interaction with tumor volume and season. All statistically significant effects plotted. Transparent dots are raw data, shaded area is confidence intervals.

**Figure 4:**
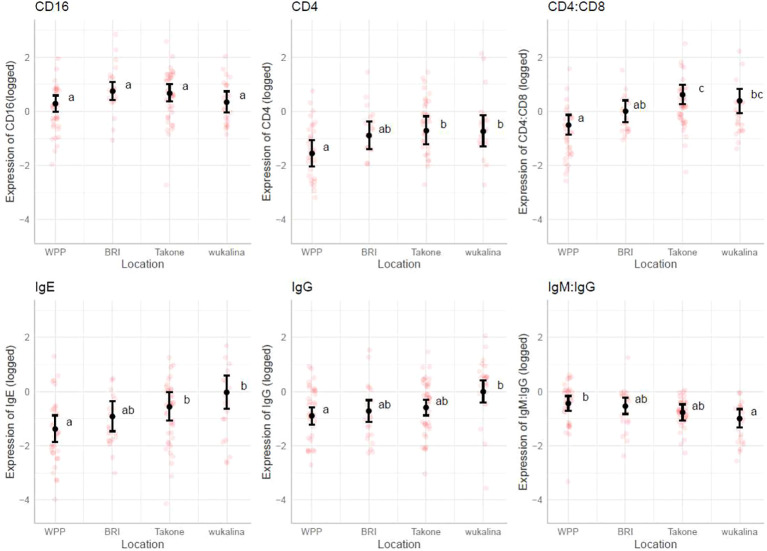
Marginal means plots, immune gene expression with location. All statistically significant results plotted, transparent dots represent raw data, confidence intervals displayed. Letters represent statistically significant differences between groups, measured using Sidak post-hot test.

The marginal means plots for the effects of tumor volume showed that all B-cell receptors (immunoglobulins), CD8 and NKG2D decreased in expression with an increase in tumor volume (**Figure 3**), while both CD11 and CD16 increased with tumor volume. CD16 showed a stronger increase in expression in summer, especially compared with winter. The IgM: IgG ratio decreased with increasing tumor volume, showing a stronger decrease in colder months, autumn and winter. Marginal means plots for season as a main effect are shown in **Supplementary Figure S2** and the marginal means plots for three gene models and both ratio models showed significant effects of location on gene expression (**Figure 4**).

### 3.3 Immune gene expression patterns prior to and after DFTD infection

The paired T-tests revealed that the expression of three immunoglobulin genes (IgE, IgG, IgM but not IgA) were significantly different within individuals, before and after DFTD infection (**Supplementary Figure S3**). The expression of IgE, IgG, IgM, NKG2D and the IgM : IgG ratio decreased once the devils had become infected, while it increased for CD16.

### 3.4 Can immune gene expression patterns in juvenile devils predict DFTD susceptibility?

**Figure 5:**
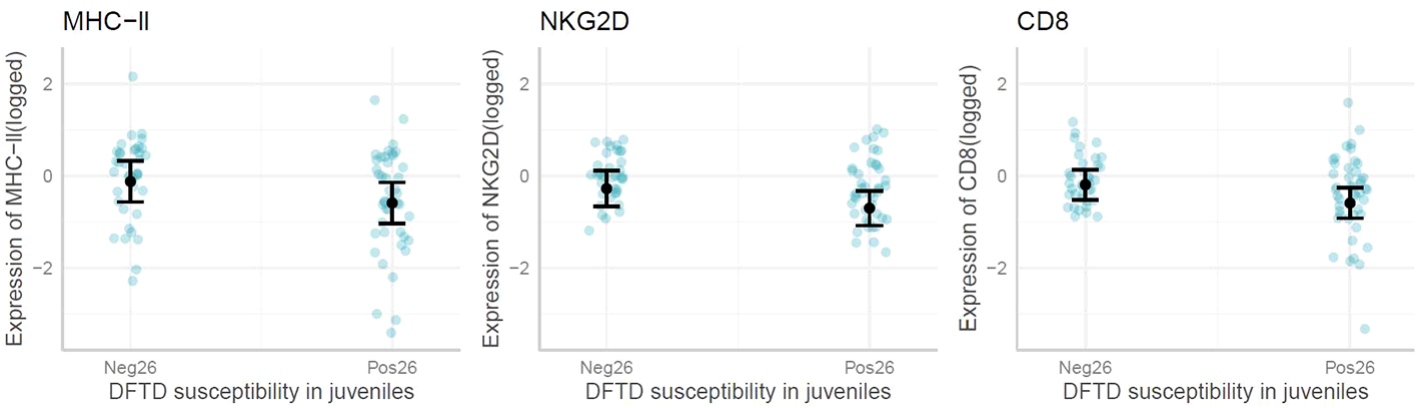
Marginal means plots showing the difference in immune gene expression between juvenile devils that present with Devil Facial Tumor Disease (DFTD) tumors by 26 months (Pos26) vs after 26 months (Neg26). Blue dots are raw data, confidence intervals displayed. Only significant results reported.

When exploring immune gene expression in juvenile animals that later displayed DFTD symptoms (by 26 months of age or later), the linear models and marginal means plots revealed no associations between the expression of most genes and early DFTD appearance (**Figure 5**). However, CD8, NKG2D and MHC-II, were significantly lower expressed (p-value <0.05) on average in young devils which later became infected with DFTD by the age of 26 months. All model outputs are presented in **Supplementary Table S2**.

#### 3.5 Immune gene expression variation throughout tumor growth and regression in a single devil

**Figure 6:**
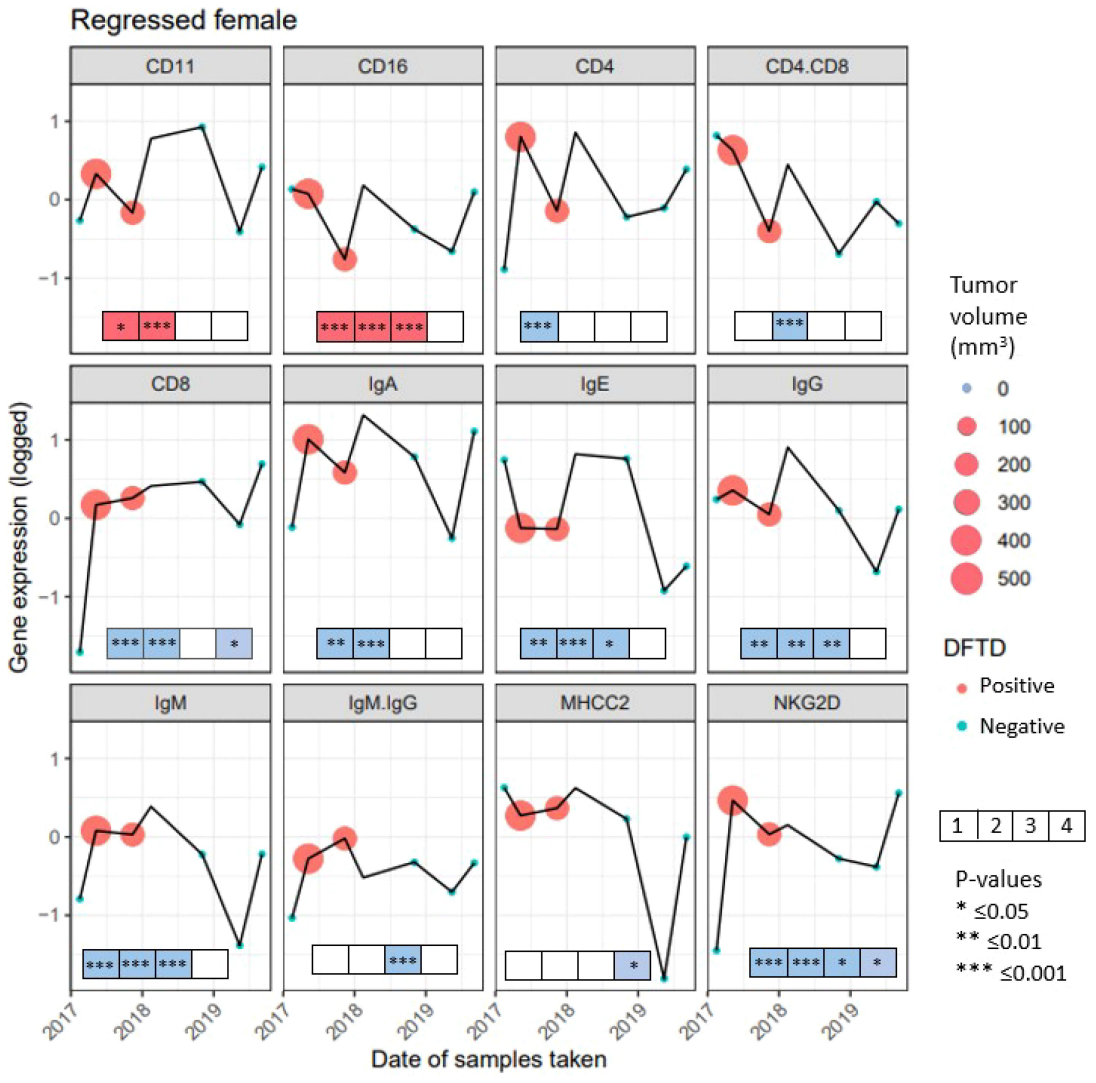
Gene expression from 10 genes and 2 gene ratios for 7 recaptures of a female devil with naturally regressed tumors. Gene names are listed above each panel, recaptures when the female had Devil Facial Tumor Disease (DFTD) are represented in red, recaptures with no visible DFTD are presented in blue. The size of the circle for captures 2 and 3 represent the tumor volume in mm^3^, the fourth capture has no color as there was no visible tumor, but the female devil was still classified as DFTD positive. To illustrate the immune gene expression patterns observed in the devil with a regressed tumor compared to the significant findings observed across the species, boxes were included at the bottom of each panel. The boxes summarize the results from the previous analyses in this study from the four analyses: First box shows the condensed results when comparing cross-sectional and longitudinal immune gene expression (see **Table 2**). Second box shows the condensed outcome when analyzing factors affecting immune gene expression in DFTD affected devils (see **Figures 2**, **3**). Third box summarizes the analysis of immune gene expression patterns prior to and after DFTD infection (see **Supplementary Figure S3**). The fourth box summarizes results of whether immune genes expression patterns in juvenile devils can predict DFTD susceptibility (see **Figure 5**) Significant results from these four previous analyses are indicated with an asterisk. Red color of the boxes indicates increase, while blue color indicates decrease in gene expression when infected with DFTD in these four previous analyses. White boxes indicate non-significant results.

We followed the expression of immune genes in a female devil that was recaptured seven times. At the first capture, the animal showed no signs of DFTD but by the second recapture the devil had a tumor with a volume of 548.38 mm^3^, that decreased by the third capture to 292.15 mm^3^. By the fourth capture the tumor was too small to be measured, but the animal was still classified as DFTD positive. After that it was listed as healthy at all remaining captures (**Figure 6**). While the expression of individual genes showed various fluctuations over time, generally it was lower at first capture when the devil did not show signs of DFTD (except CD16, IgE and MHC-II). The expression of most genes was higher at the second capture when the devil had the highest tumor volume. The expression of CD11, CD16, CD4, IgA, IgE, IgG, IgM, NKG2D decreased, while the expression of CD8 and MHC-II remained stable as the tumor volume decreased by the third capture. All genes except IgM : IgG ratio then increased expression between the third capture and when the devil cleared the tumor at the fourth capture. These results show changing patterns throughout the regression, although surprisingly most genes show the same trend, with overall increases in gene expression when the animal is infected. This pattern is different to those observed across the devils in the previous analyses, where both the cross-sectional and longitudinal data showed that most immune genes were lower expressed in animals that had DFTD, and gene expression decreased as the tumor volume increased. To demonstrate the complex individual variations in tumor progression and immune responses over time, in the **Supplementary Material** (**Supplementary Figure S4**) we present data on immune gene expression shifts and changes in tumor volume over time in Tasmanian devils captured four or more times.

## 4 Discussion

Targeted gene approaches and analyses of cross-sectional data have previously shown distinct changes in gene expression with DFTD infection. Expression of key adaptive immune genes decreased, and expression of some innate immune genes increased (21), whilst the T-cell repertoire constricted markedly (22). In contrast, a study using full transcriptomics showed little gene expression difference between infected and healthy animals (20). Our approach used both cross-sectional and longitudinal datasets, with a focus on DFTD infection, to allow insight into how individuals change their immune gene expression over the course of their life, particularly when confronted with DFTD infection. Our analyses also showed that immune gene expression patterns observed across devils were similar to those within an individual.

In previous analyses, season was significant in 75% of the gene models (21) and location was significant in >80% of gene models (Raven et al. submitted). The analyses with only DFTD-infected animals indicated reduced importance of season and location, but also reduced importance of other factors such as sex and age. These findings show that DFTD infection changes general immune patterns across both location and season, which could indicate a direct or indirect reaction to DFTD in Tasmanian devils. Our findings support a previous study that found genetic-environmental associations to abiotic factors in devils prior to DFTD, patterns that were reduced after DFTD arrived in a population (27). The authors assumed that selection for DFTD survival swamped the influence of other selective forces. Interestingly, in our study we still observed seasonal effects on the expression of both CD16 and IgM : IgG ratio with tumor volume. Seasonal influences on these genes have been found previously (21) and could indicate that the ability of devils to combat DFTD infection varies throughout the year.

Using a combination of longitudinal and cross-sectional datasets allowed us to demonstrate that immune gene expression begins to shift early in DFTD infection, and these changes in gene expression continue to become more pronounced as the tumors grow. Previous studies have shown that devils, particularly females, appear to tolerate DFTD until the tumors reach 3% of the devil’s body weight, when the disease begins to significantly impact on body condition (14). While the results of this study do not contradict the findings of the previous study, it does suggest that DFTD has gene-level effects on devils much earlier during the disease, even if it is not evidenced through changes in body condition. With disease progression, the expression pattern of more genes starts to vary, potentially indicating a direct or indirect response to DFTD. In addition to the size of the tumors, the changes in immune gene expression may be impacted by DFTD metastases. Although DFTD is a metastatic cancer, spreading from devil to devil, the conditions required within the primary tumor to spread from the site of infection to various parts of the body remains an unknown. Impact on host immune profiles from the development between primary and metastatic DFTD is also undocumented. Regardless, the increase in total tumor volume caused by the addition of internal DFTD metastases would likely impact immune profiles and therefore immune gene expression.

Here we show downregulation of immunoglobulins and upregulation of CD16 during early disease progression, indicating DFTD can impact devils earlier in infection than previously reported, and that this impact magnifies with tumor growth. Downregulation of the immunoglobulin expression could be evidence of limited immunopathology, an additional indication could be the constriction of T-cell receptors with DFTD infection, observed by Cheng et al. (22). The upregulation of CD16 and CD11 may be indicative of inflammation (51, 52), also a component of wound healing (53, 54) and a tolerance mechanism in humans (55). Wound healing is believed to be upregulated in Tasmanian devils, (56) and other studies have suggested Tasmanian devils could use tolerance as defense strategy to cope with DFTD (14). However, active immune responses (10), and tumor regression in devils (11), indicate multiple divergent defense strategies between individuals, a phenomenon observed in populations of other species (17). For example, simultaneous resistance and tolerance strategies have been detected in frogs (57), and in field voles where resistance shifted to tolerance with maturity (58). While tolerance and resistance strategies were not tested in this study, the co-existence of both could explain the study results. Additionally, Tasmanian devils show clear signs of aging-associated immune gene expression changes (21, 22, 26, 59). If these changes indicate shifts in immune strategies, it could explain why a small number of devils demonstrate DFTD reinfection when older, following tumor regression (10). The flexible strategies of Tasmanian devils to manage DFTD infection could also be facilitated, in part, by the reduction in devil population density allowing access to additional resources (60); as host immune responses have been shown to be resource dependent in other species (61, 62).

The decreasing expression of B-cell receptor genes (immunoglobulins) as well as T-cell receptor CD8 and natural killer cell receptor NKG2D with increasing tumor volume, may indicate an increasing tumor burden on devils and/or the weakening capacity to respond to the advancing disease. Patterns of higher immune gene expression in honeybees and stronger immune responses to pathogens were associated with increased wintering survival (63). However (64) showed that, on average, across several wildlife species (both invertebrates and vertebrates), less susceptible individuals had lower and more stable immune gene expression and sometimes better pathogen tolerance. It is possible that the decrease in immune gene expression with increasing tumor size in Tasmanian devils may also impair their ability to combat additional pathogens and parasites or additional immune stressors [but see (65)]. It is important to note that the observed changes in immune gene expression with increases in tumor volume are associations, not necessarily causalities. Consequently, the present study could not determine if DFTD is causing the lower immune gene expression, or if devils, rather than resisting the cancer, are suppressing their immune function to tolerate DFTD.

Individual differences in susceptibility to DFTD infection could be linked to genetic and immune gene expression variations. (24) found an association between shorter telomeres and increased DFTD infection [but see (25)], and (26) proposed that lower IgM : IgG antibody ratios increased the odds of devils becoming infected. However, the results of this study are the first to suggest that devils with lower immune gene expression as juveniles may be more prone to infection earlier in life. Out of all the genes tested, only CD8, NKG2D and MHC-II were, on average, expressed at lower levels in susceptible individuals, despite other immune genes showing changes with DFTD infection. CD8+ T-cells are cytotoxic and directly involved in detecting and destroying tumor cells (66), while NKG2D promotes tumor surveillance (67) through expression on both NK cells and CD8+ T-cells (68). As both CD8 and NKG2D are expressed on CD8+ T-cells, this may be the cell underlying the change in gene expression that was detected. MHC-II interacts with CD4+ T-cells, which drive anti-tumor responses, mostly (although not always) through regulation, making them more likely to be involved in early tumor detection (69). Reduced MHC-II expression has also been correlated with reduced tumor surveillance in humans (70). Other factors such as behavior (4), social standing (9, 35) and age (22) can also influence when a devil becomes infected with DFTD. Nevertheless, this is the first study to demonstrate that low expression of genes involved in antigen presentation when young could predispose devils to earlier DFTD manifestation.

Although we only had the opportunity to follow immune gene expression in the blood of a single devil exhibiting tumor regression, some interesting patterns emerged. The increase in gene expression with the first detection of DFTD across most of the genes was an opposing trend to those observed across devils where the expression of the studied genes generally decreases with DFTD infection (21, 22). In addition, the expression of CD16 was downregulated in this animal during the two samplings when it had visible DFTD tumors, also an opposing trend than previously observed by (21). At the fourth time point, where the animal was still classified as DFTD positive but without visible signs of tumors, every gene was upregulated, again displaying not only a different pattern to (21), but the same consistent trend across 10 genes. Although any conclusion from a single animal must be taken with caution, the coordination across the genes and the upregulation of adaptive immune genes that have been generally downregulated in DFTD affected devils, could also indicate an immune response to some (possibly independent) unmeasured variables. Human studies on natural tumor regressions show association with microbe activity within tumors (71), and the immune response evoked by specific microbes has been proposed to unveil hiding tumor cells, due to the evidence showing the subsequent clearing and recovery of even malignant tumors (72). Cancer models have shown that bacteria can colonize and potentially lyse tumors, stimulating the immune system (reviewed in 73). Specific bacterial immunotherapy can result in enhanced tumor-specific CD4 and CD8 T cell response, leading to T cell-dependent tumor immunity and sometimes even long-term tumor specific immunity (74). While secondary infections in DFTD tumors have been reported (75), no data is available on the different infecting microbes, and thus, investigating differences or similarities between microbes infecting DFTD tumors, particularly tumors that regress, warrant further studies.

A notable caveat of our study is that with any analysis involving DFTD, it is possible that some of the healthy animals in the analysis were infected with DFTD, but not symptomatic. The DFTD latency period is estimated to average between ~3 and 9 months (76), but without a biomarker, it’s not possible to determine if an animal has been infected prior to the appearance of tumors. This means by the time external symptoms are apparent the disease might have already been affecting the devils for months. It also means that some devils classified as healthy at the time point of sampling, might already be diseased, reducing the difference in immune gene expression between sampling times or groups. For example, if the samples used in the T-test were further apart in time, it could have reduced the possibility of DFTD latency in some devils, but would have introduced other confounding variables, including larger age and tumor burden effects. In addition, without autopsy, it is currently not possible to detect internal metastasis in wild Tasmanian devils, therefore tumor burdens may be underestimated in some animals.

## 5 Conclusions

This study shows that DFTD can affect immune gene expression early in infection with downregulation of immunoglobulins and upregulation of CD16. These immune gene expression changes continue with the progression of DFTD, indicating the tumors’ continued impact on devil immune function as the disease unfolds. The effect of environment is muted, and the effect of tumors is enhanced on the expression of immune genes in animals with DFTD. No significant difference was detected in how environmental factors influenced immune gene expression within individuals, as between individuals. Juveniles with lower expression of MHC-II, NKG2D and CD8 may be more susceptible to earlier infection of DFTD, possibly due to reduced immune and tumor surveillance. A single regressed animal showed possible signs of an overall immune response that correlates with the reduction in tumor size. While this study discovered many novel aspects of how DFTD affects immune gene expression in Tasmanian devil blood, there is still much we don’t understand about this unique disease, suggesting further studies are needed to elucidate how DFTD underpins devil immune responses.

## Data availability statement

The original contributions presented in the study are included in the article/**Supplementary Materials**, further inquiries can be directed to the corresponding author/s.

## Ethics statement

The animal study was approved by Deakin University’s Animal Ethics Committee. The study was conducted in accordance with the local legislation and institutional requirements.

## Author contributions

NR: Conceptualization, Data curation, Formal analysis, Funding acquisition, Investigation, Methodology, Project administration, Visualization, Writing – original draft, Writing – review & editing. MK: Conceptualization, Data curation, Formal analysis, Methodology, Supervision, Visualization, Writing – review & editing. TM: Conceptualization, Data curation, Formal analysis, Methodology, Writing – review & editing. MJ: Data curation, Funding acquisition, Resources, Writing – review & editing. DH: Data curation, Methodology, Resources, Writing – review & editing. MR-A: Data curation, Resources, Writing – review & editing. FT: Conceptualization, Funding acquisition, Resources, Supervision, Writing – review & editing. RH: Conceptualization, Data curation, Formal analysis, Funding acquisition, Methodology, Project administration, Resources, Supervision, Writing – review & editing. BU: Conceptualization, Data curation, Formal analysis, Funding acquisition, Investigation, Methodology, Project administration, Resources, Supervision, Visualization, Writing – original draft, Writing – review & editing.
