# Supplementary material for: Complex associations between cancer progression and immune gene expression reveals early influence of transmissible cancer on Tasmanian devils
Source: Front Immunol. 2024 Mar 7;15:1286352. doi: 10.3389/fimmu.2024.1286352 (PMC10954821; doi:10.3389/fimmu.2024.1286352)
Supplement: Supplementary file 1 [file DataSheet_1.docx]

Supplementary Material

**Table S1**: Brief description of genes used in the study, including their functions, cell types they are associated with and a selection of techniques used to detect expression in blood.

**References used in table**: **^1^**(Roitt, Brostoff, & Male, 2001), ^2^(Lodge et al., 2017), ^3^(Biswas et al., 2003) ^4^(Science, 2021), ^5^(Harvard, 2021), ^6^(Lei, Wang, Wang, & Wang, 2021), ^7^(Katzenelenbogen et al., 2020), ^8^(Dang et al., 2020), ^9^(Swirski et al., 2009)), ^10^(Solovjov, Pluskota, & Plow, 2005), ^11^(Schmid et al., 2018), ^12^(Di Fulvio & Gomez-Cambronero, 2005), ^13^(Ravetch & Perussia, 1989), ^14^(Saleh et al., 1995), ^15^(Yeap et al., 2016), ^16^(Croote, Darmanis, Nadeau, & Quake, 2018), ^17^(Schraven et al., 2021), ^18^(Scott, Wolchok, & Old, 2012), ^19^(Staff et al., 2012), ^20^(Macpherson, McCoy, Johansen, & Brandtzaeg, 2008), ^21^(Davis, Selva, Kent, & Chung, 2020), ^22^(Breedveld & van Egmond, 2019), ^23^(Heineke & van Egmond, 2017), ^24^ (Karagiannis et al., 2007), ^25^(Goldinger et al., 2015), ^26^ (Rock, Reits, & Neefjes, 2016), ^27^(Bovin et al., 2004), ^28^(Rosen et al., 2004), ^29^(Salih et al., 2003), ^30^(Raulet, Gasser, Gowen, Deng, & Jung, 2013), ^31^(López-Larrea, López-Soto, & González, 2010), ^32^(López-Soto, Huergo-Zapico, Acebes-Huerta, Villa-Alvarez, & Gonzalez, 2015), ^33^(Moretta et al., 2001).

| **Gene name (abbreviation)** | **Cell types the gene is expressed on in blood** | **Technique used to detect expression in blood** | **Protein function** |
| --- | --- | --- | --- |
| Cluster of Differentiation 4 (CD4) | All T helper cells^1^,  Small % of NK cells, macrophages^2^, neutrophils^3^ (Schmid et al., 2018) | Also see gene related page on gene cards or for all genes in table^4, 5^, scRNAseq^6^, combination scRNAseq, flow cytometry and RT-qPCR^7^ | Communicates with antigen presenting cells, in T-cells usually MHC-ll, activates a range of immune pathways and responses ultimately leading to lymphokine production, adhesion, motility and activation of T-helper cells^4^. Originally identified as a marker for CD4+ T cells^1^ |
| Cluster of Differentiation 8α (CD8) | Cytotoxic T cells^6^ | scRNAseq^6^ | Mediates cell-cell interactions with immune cells, communicates with antigen presenting cells, marker for CD8+ T cells^1,6^ |
| Cluster of Differentiation 11b or Integrin Subunit Alpha M (CD11) | Dendritic cells, Monocytes, Granulocytes, Macrophages^8^ | Flow cytometry and RT-qPCR^9^, NGS-multiple RNA datasets^8^ | Mediates leukocyte adhesion and migration, phagocytosis, cell-mediated killing, chemotaxis and cellular activation^10, 11^ |
| Cluster of Differentiation 16 or Fc Fragment of IgG Receptor IIIa (CD16) | Natural Killer cells^6^, Neutrophil subset^12^, Monocyte subset^6^ | scRNAseq^6^ using mAb^13^, Immuflorescence and PCR, neutrophil isolation and RT-qPCR^12^ | Marker for Natural Killer cells, activates the antigen dependent cytotoxicity cascade (ADCC), stimulates phagocytosis, recognises unknown tumours^1,14, 15^ |
| Immunoglobulin G (IgG) | Circulating antibodies, as well as B cells^16, 17^ | scRNAseq^16,17^ | Anti-toxin, stimulates phagocytosis, activates compliment pathway and ADCC^1^ |
| Immunoglobulin M (IgM) | Circulating antibodies, as well as B cells^16,17^ | scRNAseq^16,17^ | Responds to infectious organisms, activates complement and ADCC ^1,18^ |
| Immunoglobulin A (IgA) (serum) | Circulating antibodies, as well as B cells^16,17^ | scRNAseq^16,17^ | Neutralizes pathogens (bacteria and virus) and exotoxins, weak activator of complement, activates ADCC via neutrophils^1,19,20^ enhances phagocytosis, both pro and anti-inflammatory (depends on binding receptor)^21^, release of cytokines, immune cell recruitment and induction of necrosis^22, 23^ |
| Immunoglobulin E (IgE) | Circulating antibodies, as well as B cells^16,17^ | scRNAseq^16,17^ | Activation of allergies, parasite resistance^16^, activates ADCC and antigen dependent cytotoxicity phagatosis^1, 24^ |
| Major Histocompatibility Complex Class ll  (MHC-ll) | Antigen presenting cells^25^, both innate and adaptive, including B cells, monocytes, macrophages, and dendritic cells ^26,27^ | combination scRNAseq, flow cytometry and RT-qPCR^7^ | Presents peptides to CD4+ T cells^1, 26^ |
| Natural Killer group 2D (NKG2D) | Natural Killer cells, subset of CD8+ T cells^28^ | using cytotoxic assays, flow cytometry and mAb^28, 29^ | Binds to ligands upregulated in response to cellular stress (including malignant or infected cells)^30,31^ and stimulates cytotoxic pathways^32,33^ |


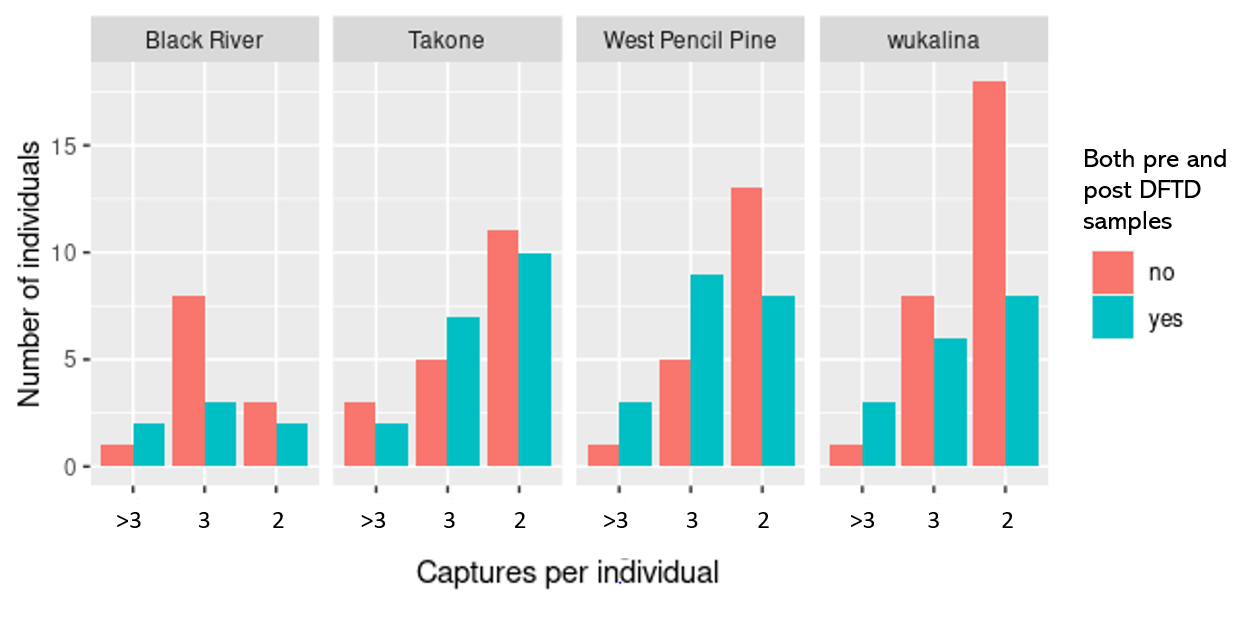


**Figure S1: Number of individual devils captured by location and number of recaptures**. Individuals grouped into two captures, three captures and more than three captures. The number of individuals with both pre and post DFTD samples highlighted in blue.


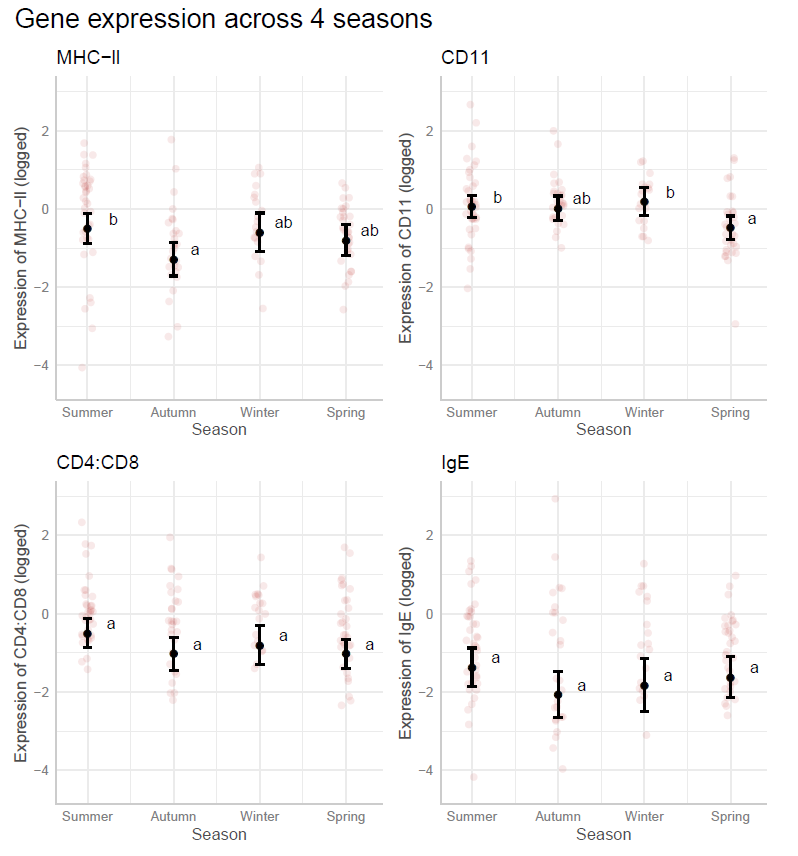


Figure S2: Marginal means plots, immune gene expression with season. All statistically significant results plotted, transparent dots = raw data, confidence intervals displayed. Letters represent statistically significant differences between groups, measured using Sidak post-hot test.


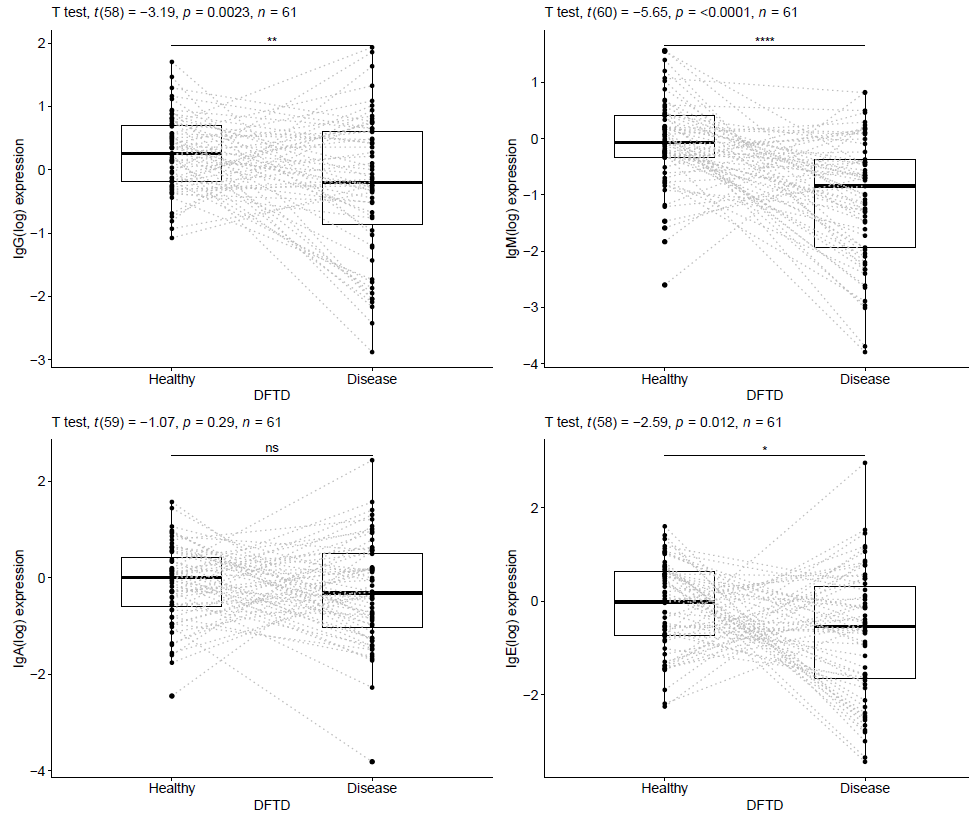


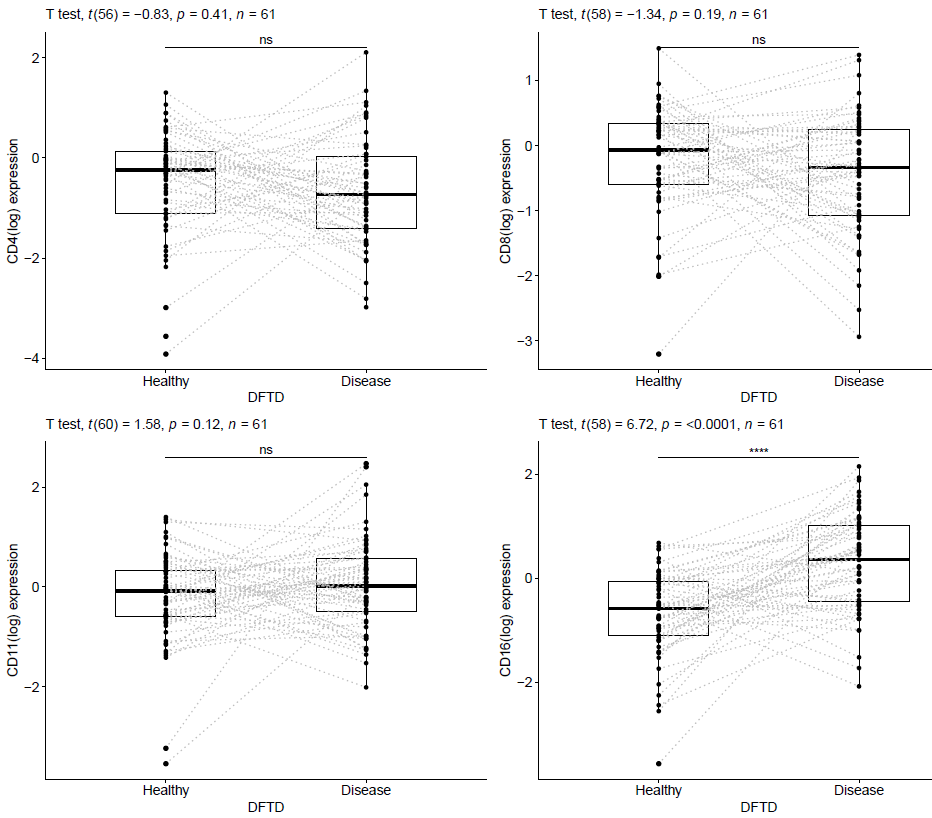


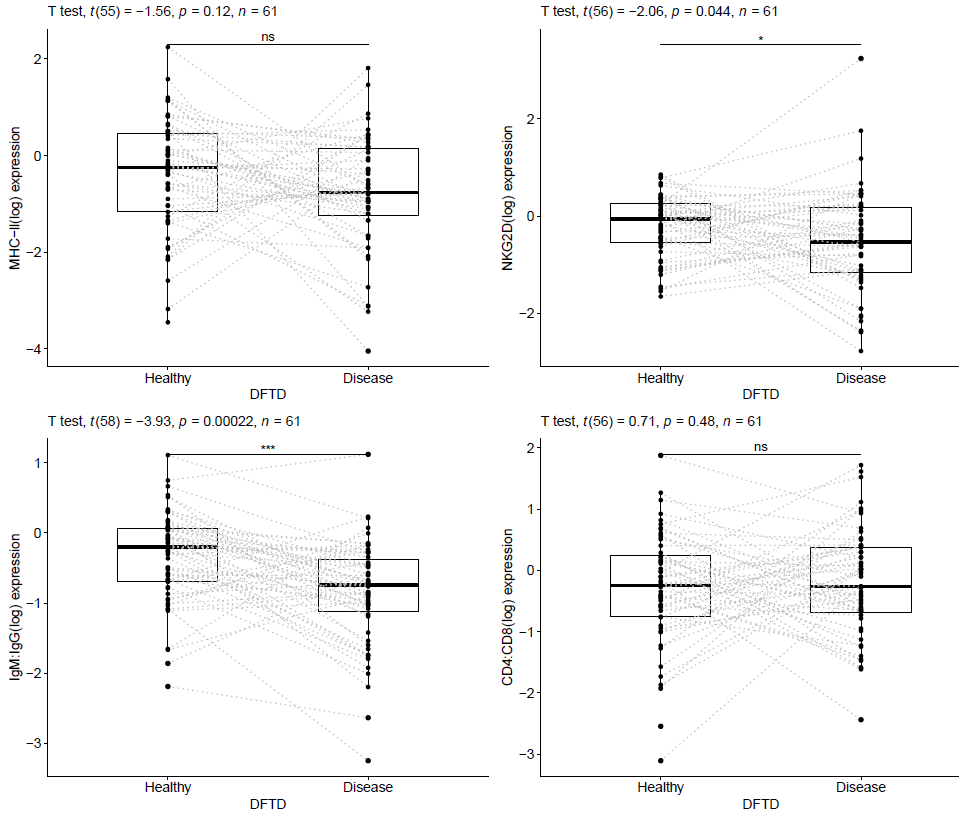


Figure S3: Paired t-tests within individuals before and after DFTD tumours. Each gene plotted separately, black dots represent raw data, grey lines link each individual pair. T-statistic, p-value and paired sample size at top of each plot. * indicate significance at p < 0.05, ** p < 0.01, *** p < 0.001.


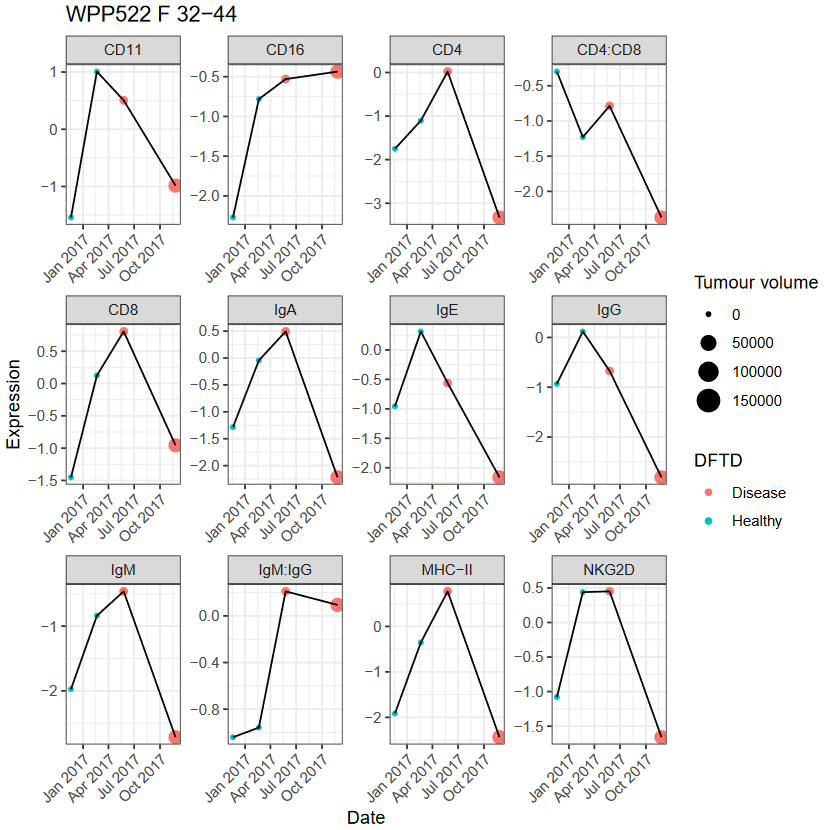


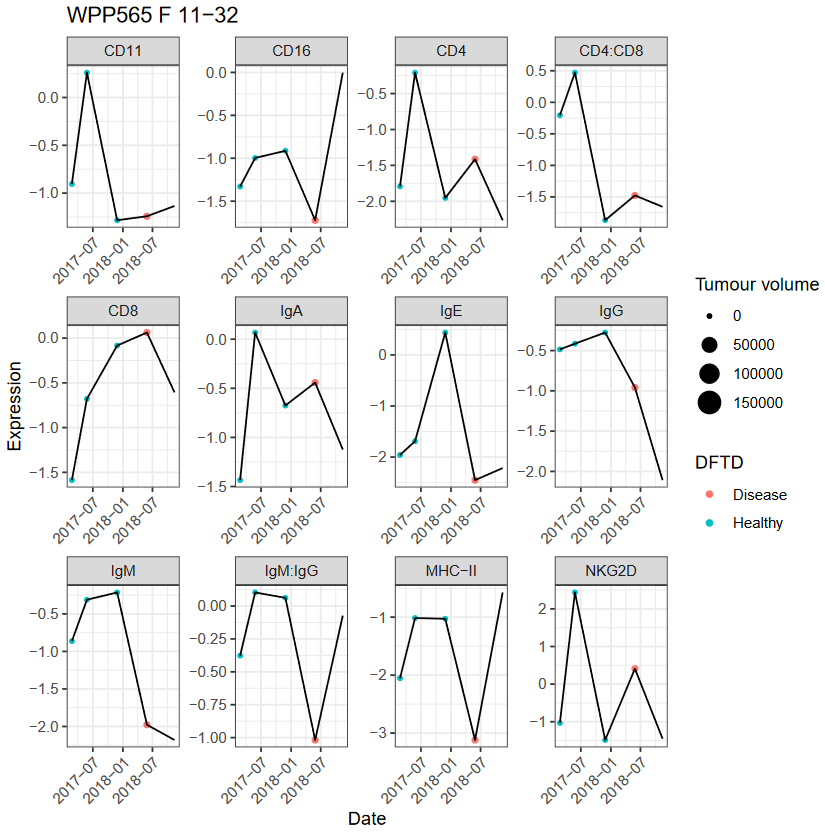


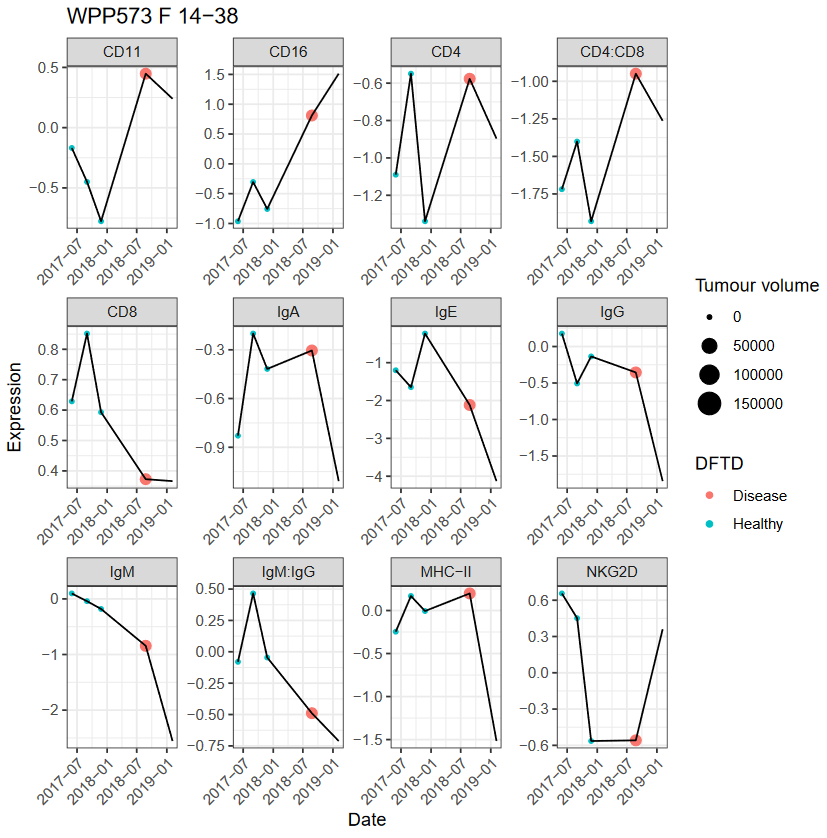


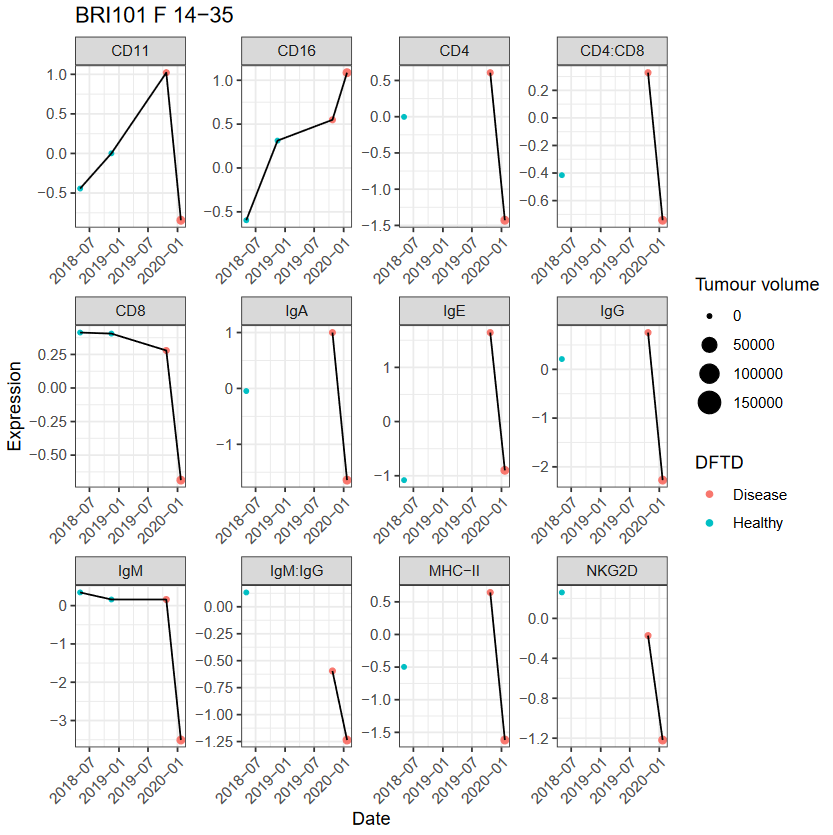


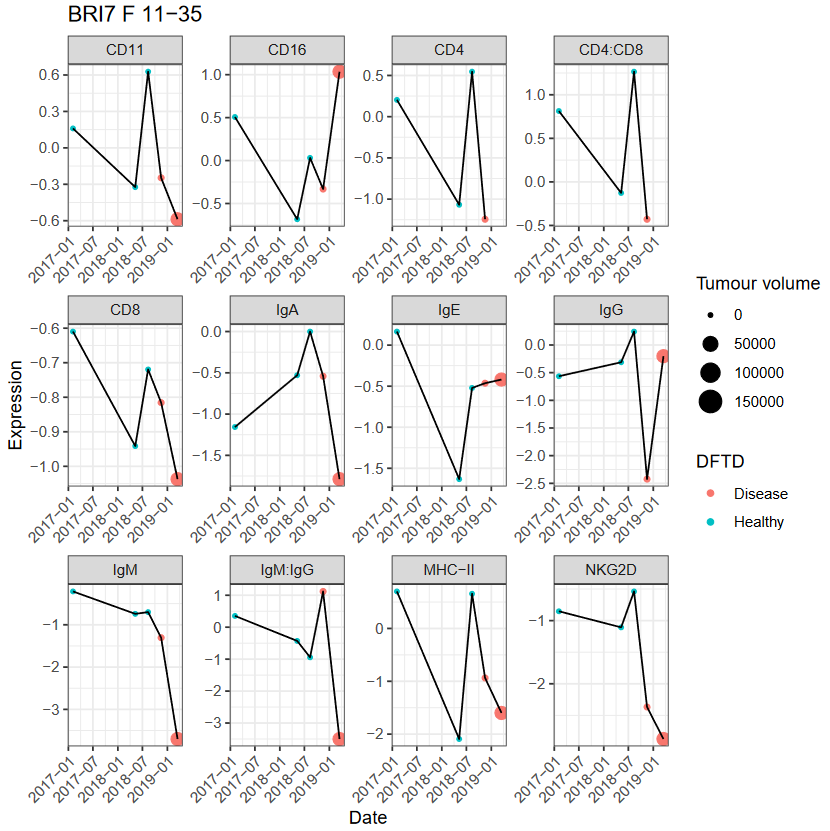


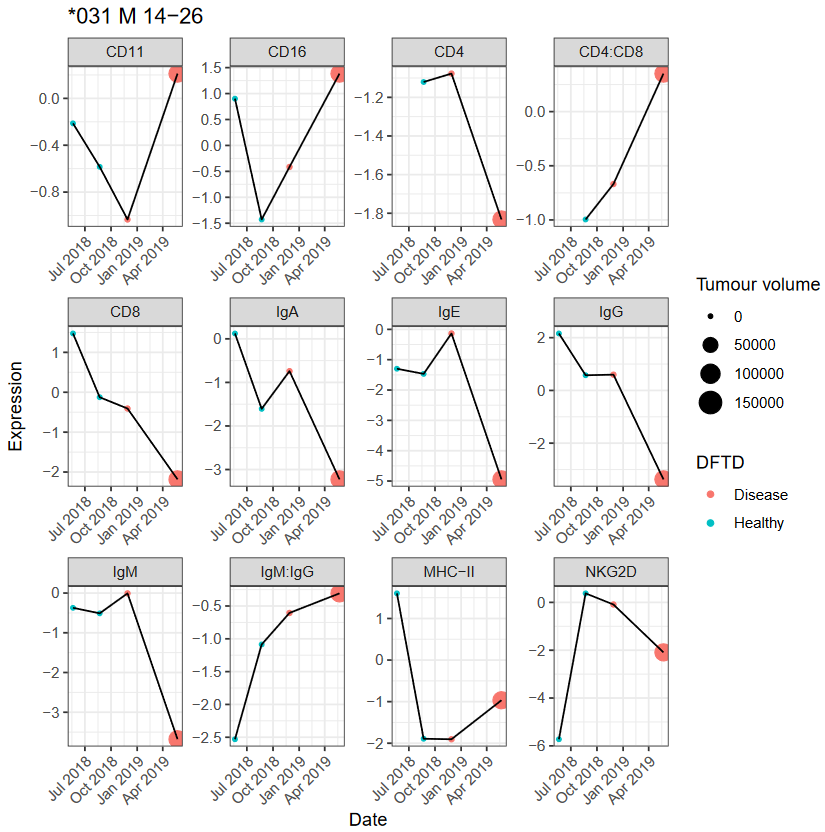


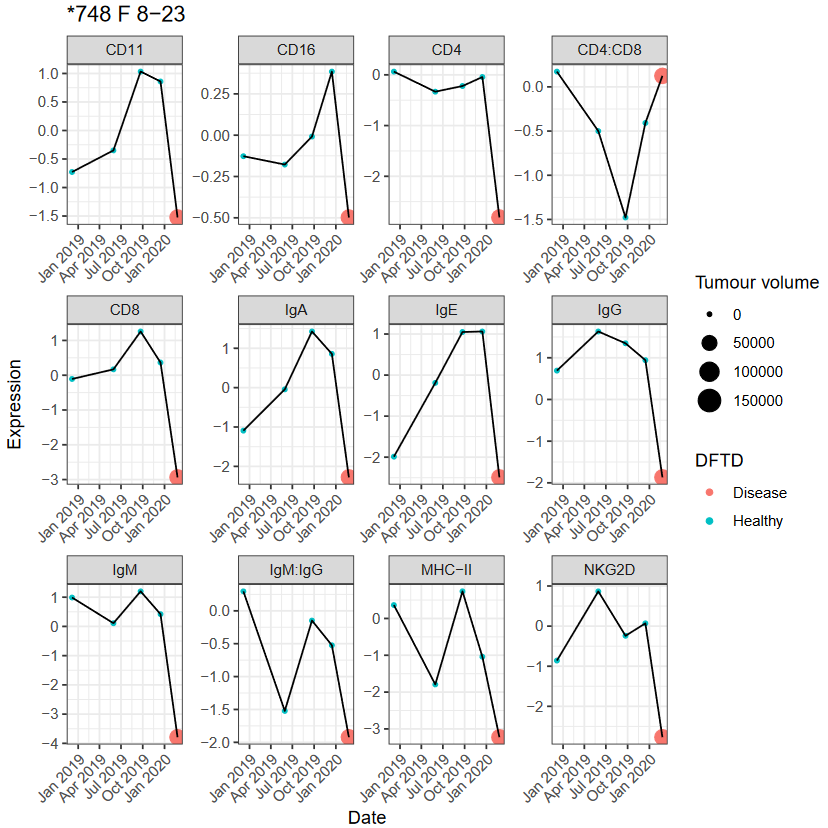


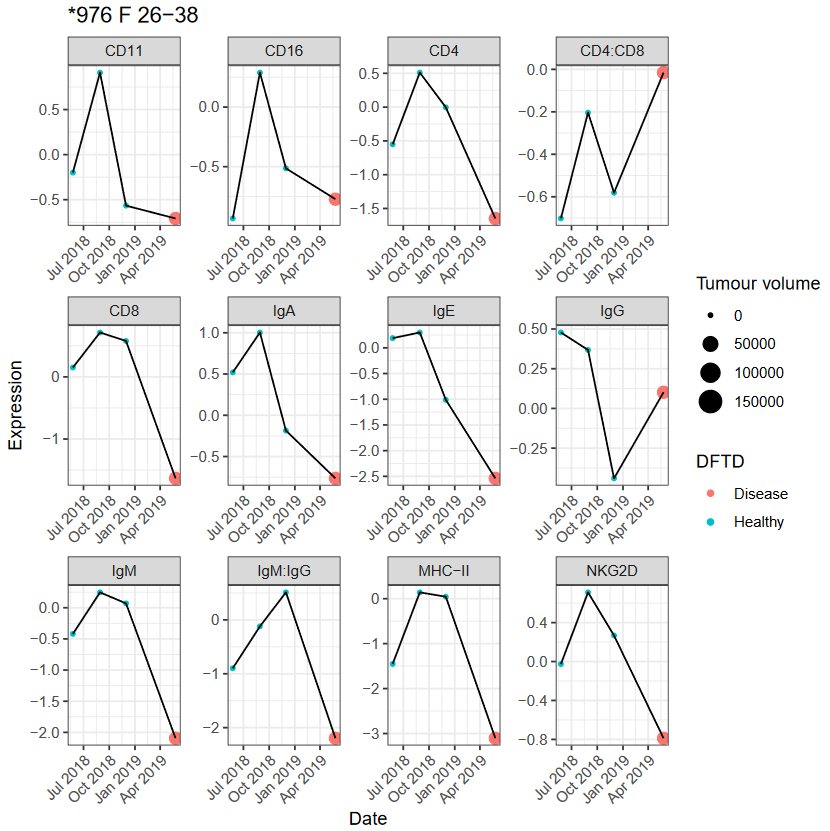


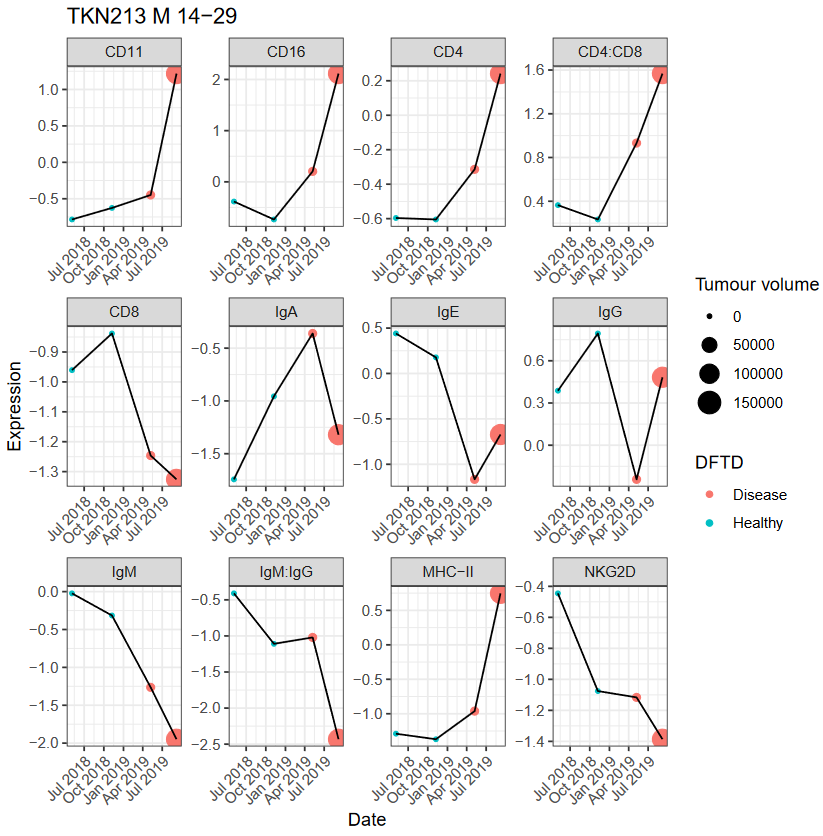


Figure S4: Complex associations between tumour volume changes and immune gene expression variations in recaptured devils.

Each panel of 12 genes is an individual devil captured four or more times. Titles contain individual devils identifier, then M (male) or F (female), followed by the age range of the individual devil, in months, across the sampling period. Gene names are listed above each panel, recaptures when the devils had Devil Facial Tumour Disease (DFTD) are represented in red, recaptures with no visible DFTD are presented in blue. The size of the circle for DFTD positive captures represent the tumour volume in mm^3^. The line joining the sampling points is broken if there is missing data.

Table S2: Model outputs for gene models using tumour volume

Model outputs for each gene analysed. Significant values in bold.

|  | **IgM linear model** | | | | |
| --- | --- | --- | --- | --- | --- |
| *Predictors* | *Estimate* | *std. Error* | *95% CI* | *t-value* | *p-value* |
| (Intercept) | -1.62 | 0.20 | -2.02 – -1.22 | -8.10 | **<0.001** |
| Tumour volume | -0.46 | 0.09 | -0.64 – -0.28 | -5.06 | **<0.001** |
| Season [Autumn] | 0.08 | 0.30 | -0.51 – 0.68 | 0.27 | 0.789 |
| Season [Winter] | 0.83 | 0.32 | 0.19 – 1.47 | 2.56 | **0.012** |
| Season [Spring] | 0.01 | 0.30 | -0.58 – 0.60 | 0.04 | 0.971 |
| Sex [M] | 0.96 | 0.28 | 0.40 – 1.51 | 3.40 | **0.001** |
| Age | -0.13 | 0.12 | -0.37 – 0.11 | -1.06 | 0.291 |
| Season [Autumn] * Sex [M] | -0.87 | 0.43 | -1.73 – -0.01 | -2.01 | **0.047** |
| Season [Winter] * Sex [M] | -1.77 | 0.49 | -2.74 – -0.80 | -3.61 | **<0.001** |
| Season [Spring] * Sex [M] | -0.50 | 0.42 | -1.34 – 0.34 | -1.18 | 0.240 |
| Sex [M] * Age | -0.22 | 0.17 | -0.55 – 0.11 | -1.31 | 0.193 |
| R^2^ / R^2^ adjusted | 0.418 / 0.364 | | | | |
| AIC | 316.465 | | | | |
|  |  | | | | |

|  | **NKG2D model** | | | |
| --- | --- | --- | --- | --- |
| *Predictors* | *Estimate* | *std. Error* | *95% CI* | *p-value* |
| (Intercept) | -0.99 | 0.22 | -1.42 – -0.56 | **<0.001** |
| Tumour volume | -0.34 | 0.09 | -0.52 – -0.15 | **<0.001** |
| Season [Autumn] | 0.27 | 0.33 | -0.39 – 0.92 | 0.422 |
| Season [Winter] | 0.76 | 0.35 | 0.06 – 1.46 | **0.032** |
| Season [Spring] | -0.34 | 0.32 | -0.97 – 0.30 | 0.294 |
| Sex [M] | 0.70 | 0.31 | 0.10 – 1.31 | **0.024** |
| Season [Autumn] * Sex [M] | -0.42 | 0.49 | -1.38 – 0.55 | 0.392 |
| Season [Winter] * Sex [M] | -1.46 | 0.53 | -2.51 – -0.41 | **0.007** |
| Season [Spring] * Sex [M] | -0.52 | 0.46 | -1.44 – 0.39 | 0.259 |
| R^2^ / R^2^ adjusted | 0.219 / 0.161 | | | |
| AICc | 333.352 | | | |

|  | **CD8 model** | | | |
| --- | --- | --- | --- | --- |
| *Predictors* | *Estimate* | *std. Error* | *95% CI* | *p-value* |
| (Intercept) | -0.94 | 0.20 | -1.33 – -0.55 | **<0.001** |
| Tumour volume | -0.28 | 0.08 | -0.45 – -0.12 | **0.001** |
| Season [Autumn] | 0.03 | 0.30 | -0.56 – 0.61 | 0.928 |
| Season [Winter] | 0.87 | 0.31 | 0.25 – 1.49 | **0.007** |
| Season [Spring] | 0.23 | 0.29 | -0.34 – 0.79 | 0.428 |
| Sex [M] | 0.77 | 0.27 | 0.23 – 1.32 | **0.006** |
| Season [Autumn] * Sex [M] | -0.32 | 0.42 | -1.16 – 0.52 | 0.452 |
| Season [Winter] * Sex [M] | -1.48 | 0.48 | -2.42 – -0.54 | **0.002** |
| Season [Spring] * Sex [M] | -0.82 | 0.41 | -1.64 – -0.00 | **0.050** |
| R^2^ / R^2^ adjusted | 0.198 / 0.139 | | | |
| AICc | 312.601 | | | |

|  | **CD4:CD8 model** | | | |
| --- | --- | --- | --- | --- |
| *Predictors* | *Estimate* | *std. Error* | *95% CI* | *p-value* |
| (Intercept) | -0.51 | 0.18 | -0.87 – -0.15 | **0.006** |
| Tumour volume | 0.14 | 0.09 | -0.03 – 0.31 | 0.100 |
| Season [Autumn] | -0.53 | 0.22 | -0.96 – -0.10 | **0.015** |
| Season [Winter] | -0.31 | 0.24 | -0.79 – 0.17 | 0.200 |
| Season [Spring] | -0.53 | 0.21 | -0.94 – -0.12 | **0.012** |
| Location [BRI] | 0.50 | 0.24 | 0.02 – 0.98 | **0.042** |
| Location [Takone] | 1.12 | 0.20 | 0.72 – 1.52 | **<0.001** |
| Location [wukalina] | 0.88 | 0.24 | 0.41 – 1.35 | **<0.001** |
| Age | 0.10 | 0.09 | -0.09 – 0.28 | 0.306 |
| R^2^ / R^2^ adjusted | 0.306 / 0.253 | | | |
| AICc | 295.315 | | | |

|  | **IgA model** | | | |
| --- | --- | --- | --- | --- |
| *Predictors* | *Estimate* | *std. Error* | *95% CI* | *p-value* |
| (Intercept) | -0.53 | 0.09 | -0.70 – -0.36 | **<0.001** |
| Tumour volume | -0.34 | 0.09 | -0.51 – -0.17 | **<0.001** |
| R^2^ / R^2^ adjusted | 0.117 / 0.110 | | | |
| AICc | 327.136 | | | |

|  | **IgE model** | | | |
| --- | --- | --- | --- | --- |
| *Predictors* | *Estimate* | *std. Error* | *95% CI* | *p-value* |
| (Intercept) | -1.37 | 0.25 | -1.86 – -0.87 | **<0.001** |
| Tumour volume | -0.56 | 0.11 | -0.78 – -0.34 | **<0.001** |
| Location [BRI] | 0.46 | 0.33 | -0.20 – 1.11 | 0.173 |
| Location [Takone] | 0.83 | 0.28 | 0.26 – 1.39 | **0.004** |
| Location [wukalina] | 1.35 | 0.33 | 0.69 – 2.01 | **<0.001** |
| Season [Autumn] | -0.70 | 0.31 | -1.31 – -0.09 | **0.026** |
| Season [Winter] | -0.45 | 0.34 | -1.12 – 0.22 | 0.182 |
| Season [Spring] | -0.25 | 0.29 | -0.83 – 0.32 | 0.385 |
| R^2^ / R^2^ adjusted | 0.300 / 0.256 | | | |
| AICc | 390.875 | | | |

|  | **IgG model** | | | |
| --- | --- | --- | --- | --- |
| *Predictors* | *Estimate* | *std. Error* | *95% CI* | *p-value* |
| (Intercept) | -0.86 | 0.16 | -1.18 – -0.54 | **<0.001** |
| Tumour volume | -0.25 | 0.09 | -0.44 – -0.07 | **0.008** |
| Location [BRI] | 0.18 | 0.26 | -0.34 – 0.71 | 0.494 |
| Location [Takone] | 0.31 | 0.22 | -0.13 – 0.75 | 0.165 |
| Location [wukalina] | 0.90 | 0.26 | 0.39 – 1.41 | **0.001** |
| Age | -0.25 | 0.10 | -0.45 – -0.06 | **0.011** |
| R^2^ / R^2^ adjusted | 0.260 / 0.227 | | | |
| AICc | 331.915 | | | |

|  | **IgM:IgG model** | | | |
| --- | --- | --- | --- | --- |
| *Predictors* | *Estimate* | *std. Error* | *95% CI* | *p-value* |
| (Intercept) | -0.43 | 0.14 | -0.71 – -0.16 | **0.002** |
| Tumour volume | -0.08 | 0.12 | -0.33 – 0.16 | 0.491 |
| Season [Autumn] | -0.43 | 0.16 | -0.76 – -0.11 | **0.009** |
| Season [Winter] | -0.28 | 0.17 | -0.62 – 0.06 | 0.101 |
| Season [Spring] | -0.04 | 0.15 | -0.34 – 0.25 | 0.781 |
| Location [BRI] | -0.09 | 0.17 | -0.43 – 0.25 | 0.604 |
| Location [Takone] | -0.33 | 0.15 | -0.63 – -0.04 | **0.026** |
| Location [wukalina] | -0.55 | 0.17 | -0.90 – -0.21 | **0.002** |
| Sex [M] | 0.24 | 0.11 | 0.01 – 0.46 | **0.040** |
| Tumour volume * Season [Autumn] | -0.44 | 0.19 | -0.82 – -0.05 | **0.026** |
| Tumour volume * Season [Winter] | -0.16 | 0.16 | -0.48 – 0.17 | 0.336 |
| Tumour volume * Season [Spring] | -0.02 | 0.16 | -0.34 – 0.29 | 0.882 |
| R^2^ / R^2^ adjusted | 0.356 / 0.290 | | | |
| AICc | 232.581 | | | |

|  | **MHC-ll model** | | | |
| --- | --- | --- | --- | --- |
| *Predictors* | *Estimate* | *std. Error* | *95% CI* | *p-value* |
| (Intercept) | -0.45 | 0.19 | -0.84 – -0.07 | **0.022** |
| Tumour volume | 0.12 | 0.11 | -0.09 – 0.33 | 0.246 |
| Season [Autumn] | -0.79 | 0.26 | -1.30 – -0.27 | **0.003** |
| Season [Winter] | -0.09 | 0.29 | -0.66 – 0.48 | 0.747 |
| Season [Spring] | -0.29 | 0.25 | -0.79 – 0.20 | 0.242 |
| Sex [M] | 0.39 | 0.19 | 0.01 – 0.76 | **0.044** |
| Age | -0.29 | 0.14 | -0.57 – -0.01 | **0.040** |
| Sex [M] * Age | 0.53 | 0.19 | 0.15 – 0.91 | **0.006** |
| R^2^ / R^2^ adjusted | 0.188 / 0.135 | | | |
| AICc | 347.184 | | | |

|  | **CD4 model** | | | |
| --- | --- | --- | --- | --- |
| *Predictors* | *Estimate* | *std. Error* | *95% CI* | *p-value* |
| (Intercept) | -1.56 | 0.25 | -2.05 – -1.06 | **<0.001** |
| Season [Autumn] | -0.06 | 0.33 | -0.71 – 0.59 | 0.853 |
| Season [Winter] | 0.72 | 0.36 | 0.01 – 1.44 | **0.048** |
| Season [Spring] | -0.18 | 0.31 | -0.80 – 0.44 | 0.560 |
| Location [BRI] | 0.67 | 0.26 | 0.15 – 1.19 | **0.012** |
| Location [Takone] | 0.85 | 0.22 | 0.41 – 1.29 | **<0.001** |
| Location [wukalina] | 0.83 | 0.26 | 0.32 – 1.35 | **0.002** |
| Sex [M] | 0.86 | 0.31 | 0.25 – 1.47 | **0.006** |
| Season [Autumn] * Sex [M] | -1.10 | 0.46 | -2.02 – -0.18 | **0.019** |
| Season [Winter] * Sex [M] | -1.52 | 0.53 | -2.58 – -0.47 | **0.005** |
| Season [Spring] * Sex [M] | -0.62 | 0.44 | -1.50 – 0.26 | 0.165 |
| R^2^ / R^2^ adjusted | 0.297 / 0.230 | | | |
| AICc | 320.074 | | | |

|  | **CD16 model** | | | |
| --- | --- | --- | --- | --- |
| *Predictors* | *Estimate* | *std. Error* | *95% CI* | *p-value* |
| (Intercept) | 0.30 | 0.15 | -0.01 – 0.60 | 0.055 |
| Tumour volume | 0.77 | 0.15 | 0.48 – 1.07 | **<0.001** |
| Season [Autumn] | -0.06 | 0.20 | -0.44 – 0.33 | 0.775 |
| Season [Winter] | 0.16 | 0.20 | -0.24 – 0.56 | 0.432 |
| Season [Spring] | -0.46 | 0.18 | -0.81 – -0.10 | **0.011** |
| Location [BRI] | 0.47 | 0.20 | 0.07 – 0.87 | **0.022** |
| Location [Takone] | 0.40 | 0.18 | 0.05 – 0.75 | **0.024** |
| Location [wukalina] | 0.06 | 0.21 | -0.35 – 0.48 | 0.759 |
| Tumour volume * Season [Autumn] | -0.36 | 0.23 | -0.81 – 0.10 | 0.126 |
| Tumour volume * Season [Winter] | -0.44 | 0.20 | -0.83 – -0.04 | **0.029** |
| Tumour volume * Season [Spring] | -0.23 | 0.19 | -0.61 – 0.15 | 0.228 |
| R^2^ / R^2^ adjusted | 0.475 / 0.426 | | | |
| AICc | 269.811 | | | |

|  | **CD11 model** | | | |
| --- | --- | --- | --- | --- |
| *Predictors* | *Estimate* | *std. Error* | *95% CI* | *p-value* |
| (Intercept) | 0.06 | 0.14 | -0.23 – 0.34 | 0.699 |
| Tumour volume | 0.25 | 0.07 | 0.10 – 0.39 | **0.001** |
| Season [Autumn] | -0.04 | 0.19 | -0.42 – 0.34 | 0.842 |
| Season [Winter] | 0.13 | 0.21 | -0.29 – 0.55 | 0.547 |
| Season [Spring] | -0.54 | 0.18 | -0.90 – -0.17 | **0.004** |
| Sex [M] | 0.18 | 0.14 | -0.10 – 0.46 | 0.200 |
| R^2^ / R^2^ adjusted | 0.207 / 0.172 | | | |
| AICc | 283.725 | | | |

Table S2: Model outputs for susceptibility gene models

Model outputs for each gene analysed. Significant values in bold.


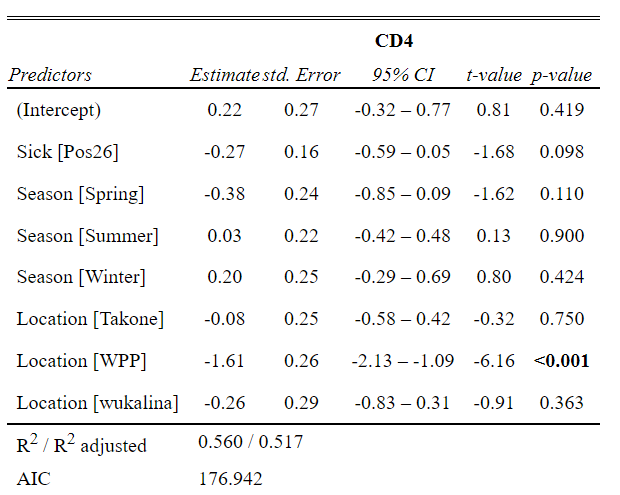


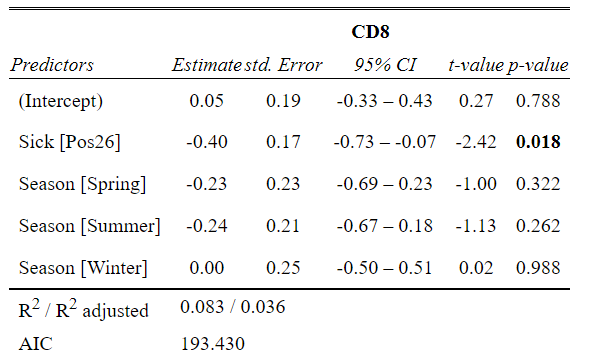


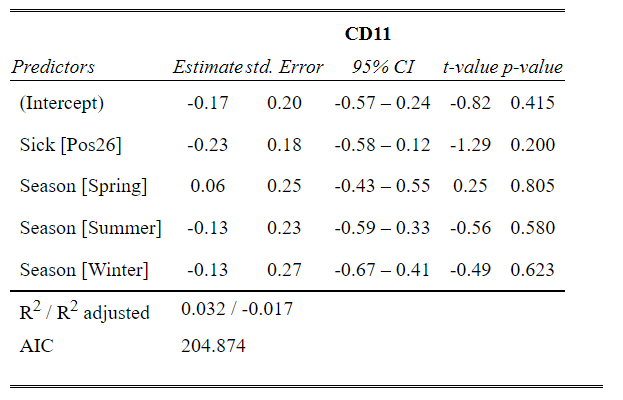


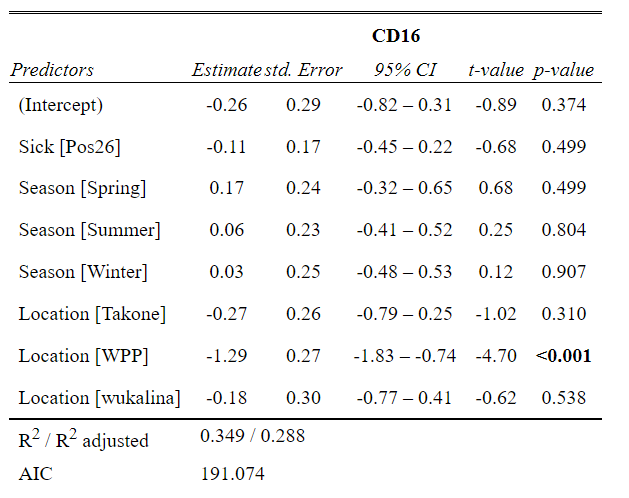


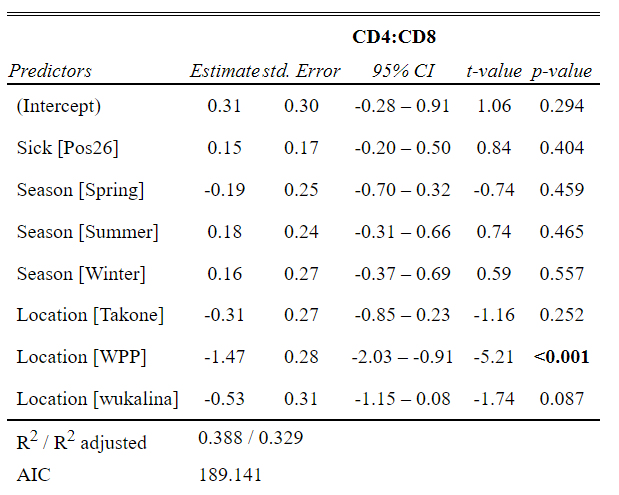


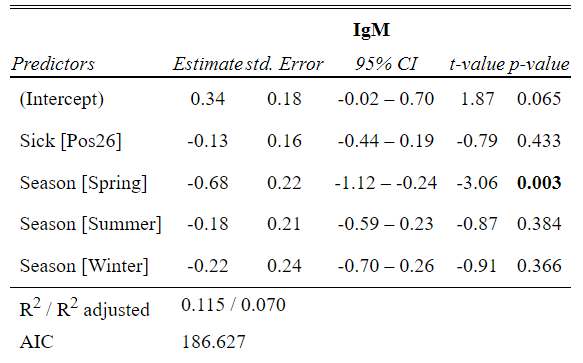


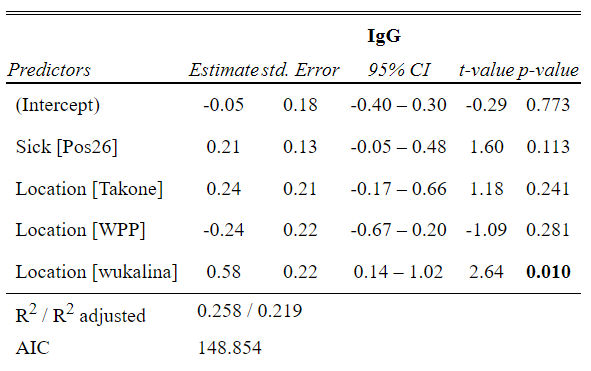


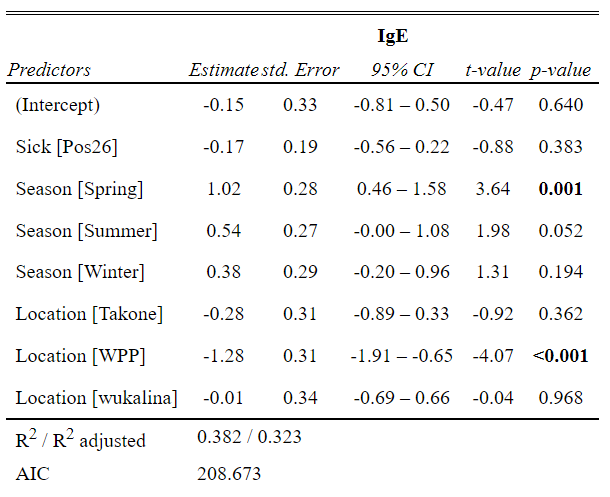


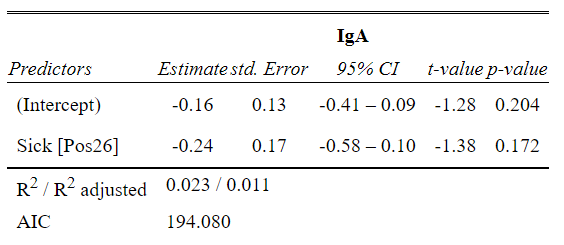


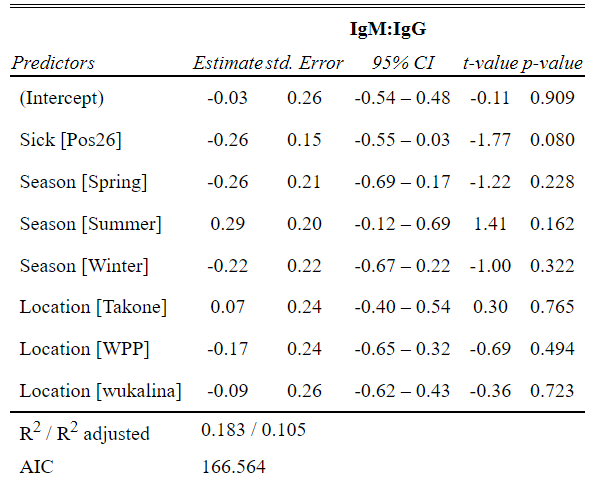


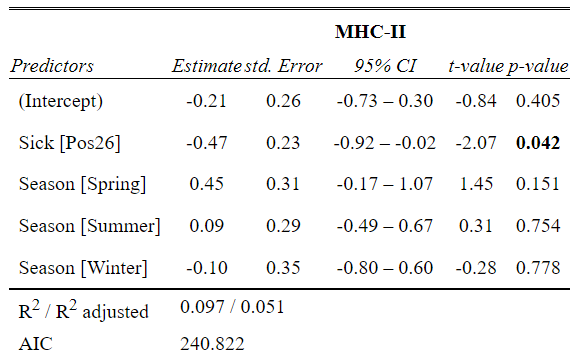


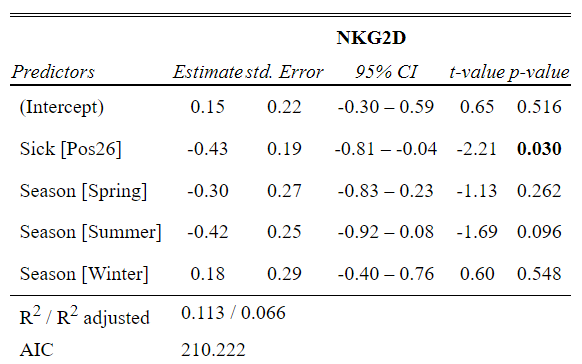


Biswas, P., Mantelli, B., Sica, A., Malnati, M., Panzeri, C., Saccani, A., . . . Beretta, A. (2003). Expression of CD4 on human peripheral blood neutrophils. *Blood, 101*(11), 4452-4456. doi:10.1182/blood-2002-10-3056 %J Blood

Bovin, L. F., Rieneck, K., Workman, C., Nielsen, H., Sørensen, S. F., Skjødt, H., . . . Bendtzen, K. (2004). Blood cell gene expression profiling in rheumatoid arthritis: Discriminative genes and effect of rheumatoid factor. *Immunology Letters, 93*(2), 217-226. doi:<https://doi.org/10.1016/j.imlet.2004.03.018>

Breedveld, A., & van Egmond, M. (2019). IgA and FcαRI: Pathological Roles and Therapeutic Opportunities. *Frontiers in Immunology, 10*(553). doi:10.3389/fimmu.2019.00553

Croote, D., Darmanis, S., Nadeau, K. C., & Quake, S. R. (2018). High-affinity allergen-specific human antibodies cloned from single IgE B cell transcriptomes. *Science, 362*(6420), 1306-1309. doi:doi:10.1126/science.aau2599

Dang, D., Taheri, S., Das, S., Ghosh, P., Prince, L. S., & Sahoo, D. J. F. i. p. (2020). Computational approach to identifying universal macrophage biomarkers. *11*, 275.

Davis, S. K., Selva, K. J., Kent, S. J., & Chung, A. W. (2020). Serum IgA Fc effector functions in infectious disease and cancer. *Immunology & Cell Biology, 98*(4), 276-286. doi:<https://doi.org/10.1111/imcb.12306>

Di Fulvio, M., & Gomez-Cambronero, J. (2005). Phospholipase D (PLD) gene expression in human neutrophils and HL-60 differentiation. *Journal of Leukocyte Biology, 77*(6), 999-1007. doi:<https://doi.org/10.1189/jlb.1104684>

Goldinger, A., Shakhbazov, K., Henders, A. K., McRae, A. F., Montgomery, G. W., & Powell, J. E. (2015). Seasonal Effects on Gene Expression. *Plos One, 10*(5), e0126995. doi:10.1371/journal.pone.0126995

Harvard, B. I. o. M. a. (2021). Gene page. Retrieved from <https://www.gtexportal.org/home/gene/CD4>

Heineke, M. H., & van Egmond, M. (2017). Immunoglobulin A: magic bullet or Trojan horse? *European Journal of Clinical Investigation, 47*(2), 184-192. doi:<https://doi.org/10.1111/eci.12716>

Karagiannis, S. N., Bracher, M. G., Hunt, J., McCloskey, N., Beavil, R. L., Beavil, A. J., . . . Gould, H. J. (2007). IgE-Antibody-Dependent Immunotherapy of Solid Tumors: Cytotoxic and Phagocytic Mechanisms of Eradication of Ovarian Cancer Cells. *The Journal of Immunology, 179*(5), 2832. doi:10.4049/jimmunol.179.5.2832

Katzenelenbogen, Y., Sheban, F., Yalin, A., Yofe, I., Svetlichnyy, D., Jaitin, D. A., . . . Amit, I. (2020). Coupled scRNA-Seq and Intracellular Protein Activity Reveal an Immunosuppressive Role of TREM2 in Cancer. *Cell, 182*(4), 872-885.e819. doi:<https://doi.org/10.1016/j.cell.2020.06.032>

Lei, H., Wang, C., Wang, Y., & Wang, C. (2021). Single-cell RNA-Seq revealed profound immune alteration in the peripheral blood of patients with bacterial infection. *International Journal of Infectious Diseases, 103*, 527-535.

Lodge, R., Ferreira Barbosa, J. A., Lombard-Vadnais, F., Gilmore, J. C., Deshiere, A., Gosselin, A., . . . Cohen, É. A. (2017). Host MicroRNAs-221 and -222 Inhibit HIV-1 Entry in Macrophages by Targeting the CD4 Viral Receptor. *Cell Reports, 21*(1), 141-153. doi:<https://doi.org/10.1016/j.celrep.2017.09.030>

López-Larrea, C., López-Soto, A., & González, S. (2010). Chapter Five - NK cell immune recognition: NKG2D ligands and stressed cells. In M. T. Lotze & A. W. Thomson (Eds.), *Natural Killer Cells* (pp. 65-77). San Diego: Academic Press.

López-Soto, A., Huergo-Zapico, L., Acebes-Huerta, A., Villa-Alvarez, M., & Gonzalez, S. (2015). NKG2D signaling in cancer immunosurveillance. *136*(8), 1741-1750. doi:10.1002/ijc.28775

Macpherson, A., McCoy, K., Johansen, F., & Brandtzaeg, P. (2008). The immune geography of IgA induction and function. *Mucosal immunology, 1*(1), 11-22.

Moretta, A., Bottino, C., Vitale, M., Pende, D., Cantoni, C., Mingari, M. C., . . . Moretta, L. J. A. r. o. i. (2001). Activating receptors and coreceptors involved in human natural killer cell-mediated cytolysis. *19*(1), 197-223.

Raulet, D. H., Gasser, S., Gowen, B. G., Deng, W., & Jung, H. (2013). Regulation of Ligands for the NKG2D Activating Receptor. *Annual review of immunology, 31*(1), 413-441. doi:10.1146/annurev-immunol-032712-095951

Ravetch, J. V., & Perussia, B. J. T. J. o. e. m. (1989). Alternative membrane forms of Fc gamma RIII (CD16) on human natural killer cells and neutrophils. Cell type-specific expression of two genes that differ in single nucleotide substitutions. *170*(2), 481-497.

Rock, K. L., Reits, E., & Neefjes, J. (2016). Present Yourself! By MHC Class I and MHC Class II Molecules. *Trends in immunology, 37*(11), 724-737. doi:<https://doi.org/10.1016/j.it.2016.08.010>

Roitt, I., Brostoff, J., & Male, D. (2001). *Immunology* (Vol. 6). London: Mosby.

Rosen, D. B., Araki, M., Hamerman, J. A., Chen, T., Yamamura, T., & Lanier, L. L. (2004). A Structural Basis for the Association of DAP12 with Mouse, but Not Human, NKG2D. *173*(4), 2470-2478. doi:10.4049/jimmunol.173.4.2470 %J The Journal of Immunology

Saleh, M. N., Goldman, S. J., LoBuglio, A. F., Beall, A. C., Sabio, H., McCord, M. C., . . . Munn, D. H. (1995). CD16+ monocytes in patients with cancer: spontaneous elevation and pharmacologic induction by recombinant human macrophage colony- stimulating factor. *Blood, 85*(10), 2910-2917. doi:10.1182/blood.V85.10.2910.bloodjournal85102910

Salih, H. R., Antropius, H., Gieseke, F., Lutz, S. Z., Kanz, L., Rammensee, H.-G., & Steinle, A. (2003). Functional expression and release of ligands for the activating immunoreceptor NKG2D in leukemia. *Blood, 102*(4), 1389-1396.

Schmid, M. C., Khan, S. Q., Kaneda, M. M., Pathria, P., Shepard, R., Louis, T. L., . . . Varner, J. A. (2018). Integrin CD11b activation drives anti-tumor innate immunity. *Nature Communications, 9*(1), 5379. doi:10.1038/s41467-018-07387-4

Schraven, A. L., Hansen, V. L., Morrissey, K. A., Stannard, H. J., Ong, O. T. W., Douek, D. C., . . . Old, J. M. (2021). Single-cell transcriptome analysis of the B-cell repertoire reveals the usage of immunoglobulins in the gray short-tailed opossum (Monodelphis domestica). *Developmental & Comparative Immunology, 123*, 104141. doi:<https://doi.org/10.1016/j.dci.2021.104141>

Science, W. I. o. (2021). GeneCards/CD4. *GeneCards.* Retrieved from <https://www.genecards.org/cgi-bin/carddisp.pl?gene=CD4&keywords=cd4#expression>

Scott, A. M., Wolchok, J. D., & Old, L. J. (2012). Antibody therapy of cancer. *Nature Reviews Cancer, 12*(4), 278-287. doi:10.1038/nrc3236

Solovjov, D. A., Pluskota, E., & Plow, E. F. (2005). Distinct roles for the α and β subunits in the functions of integrin αMβ2. *Journal of biological chemistry*

*280*(2), 1336-1345.

Staff, C., Magnusson, C. G. M., Hojjat-Farsangi, M., Mosolits, S., Liljefors, M., Frödin, J.-E., . . . Ullenhag, G. J. (2012). Induction of IgM, IgA and IgE Antibodies in Colorectal Cancer Patients Vaccinated with a Recombinant CEA Protein. *Journal of Clinical Immunology, 32*(4), 855-865. doi:10.1007/s10875-012-9662-7

Swirski, F. K., Nahrendorf, M., Etzrodt, M., Wildgruber, M., Cortez-Retamozo, V., Panizzi, P., . . . Pittet, M. J. (2009). Identification of Splenic Reservoir Monocytes and Their Deployment to Inflammatory Sites. *325*(5940), 612-616. doi:10.1126/science.1175202 %J Science

Yeap, W. H., Wong, K. L., Shimasaki, N., Teo, E. C. Y., Quek, J. K. S., Yong, H. X., . . . Wong, S. C. (2016). CD16 is indispensable for antibody-dependent cellular cytotoxicity by human monocytes. *Scientific Reports, 6*, 34310-34310. doi:10.1038/srep34310
